# Cancer- and behavior-related genes are targeted by selection in the Tasmanian devil (*Sarcophilus harrisii*)

**DOI:** 10.1371/journal.pone.0201838

**Published:** 2018-08-13

**Authors:** Jean-Noël Hubert, Tatiana Zerjal, Frédéric Hospital

**Affiliations:** GABI, INRA, AgroParisTech, Université Paris-Saclay, Jouy-en-Josas, France; University of Sydney, AUSTRALIA

## Abstract

Devil Facial Tumor Disease (DFTD) is an aggressive cancer notorious for its rare etiology and its impact on Tasmanian devil populations. Two regions underlying an evolutionary response to this cancer were recently identified using genomic time-series pre- and post-DTFD arrival. Here, we support that DFTD shaped the genome of the Tasmanian devil in an even more extensive way than previously reported. We detected 97 signatures of selection, including 148 protein coding genes having a human orthologue, linked to DFTD. Most candidate genes are associated with cancer progression, and an important subset of candidate genes has additional influence on social behavior. This confirms the influence of cancer on the ecology and evolution of the Tasmanian devil. Our work also demonstrates the possibility to detect highly polygenic footprints of short-term selection in very small populations.

## Introduction

Understanding the role of selection in the resistance against cancer should benefit from the study of animal populations. Domestic populations that develop spontaneous neoplasms similar to those encountered in humans have been increasingly investigated to unravel the complex genetic determinism of some cancers [1]. Studies that examine cancer in natural populations are, however, quite rare, despite their potential for improving our knowledge of cancer resistance mechanisms in an ecological context. Observations made in this field are limited to a small number of unique cancer evolution cases, as those described in the naked-mole rat, the elephant, and the Tasmanian devil [2]. In particular, horizontally transmitted cancers remain rarely observed events in nature, and so far they have been described in dogs [3], the Tasmanian devil [4] and several species of bivalves [5]. A well-known case of transmissible cancer is the one affecting the Tasmanian devil (*Sarcophilus harrisii*), the largest existing marsupial carnivore. This cancer, known as the Devil Facial Tumor Disease (DFTD), is characterized by a recent emergence, a high propensity to metastasize, and a mortality rate close to 100% within 12 months after infection [6,7,8,9]. The disease was first detected in north-eastern Tasmania in 1996 and has since spread across 95% of the species’ range [10] through biting injuries during social contact [11]. DFTD has induced a rapid decline and a very strong selective pressure on the Tasmanian devil–with local population losses over 90%–giving rise to serious concerns regarding the species survival on the short term [12].

DFTD has become a very well documented case study over the past decade. This amount of data is a valuable resource to better understand cancer biology and is expected to provide insight into the mechanisms underlying immunosurveillance of cancer initiation and metastatic spreading [7]. A recent analysis of Tasmanian devil genomic time-series [13] led to the identification of two regions exhibiting signatures of selection in response to DFTD which contain genes related to cancer risk and immune function in humans [14]. Here, we extend this research by using a customized maximum-likelihood method that has been shown efficient for investigating rapid selection in experimental populations when genomic time-series data are available [15]. In comparison to the approach used in [14], our method has in particular the advantage of efficiently disentangling the effects of strong selection from those of strong drift in a population-specific manner. In total, 97 genomic regions harboring 148 protein coding genes with human orthologues were identified. The functional analysis revealed that all the functionally characterized orthologues have a link with cancer and around 15% are involved in the functioning of the Central Nervous System (CNS), with about fifteen genes associated with behavioral disorders.

Our data support the view that the evolutionary response to DFTD consists in several strategies that rely on a larger range of genetic variants than previously thought. These findings should contribute to a better understanding of the ecology and evolution of both the Tasmanian devil and cancer. In particular, this should help to pinpoint some genes that may provide host populations with a “resistance”–either by improving cancer survival or by reducing the risk of infection–to a transmissible cancer.

## Results

### Signatures of selection in the Tasmanian devil genome

**Fig 1:**
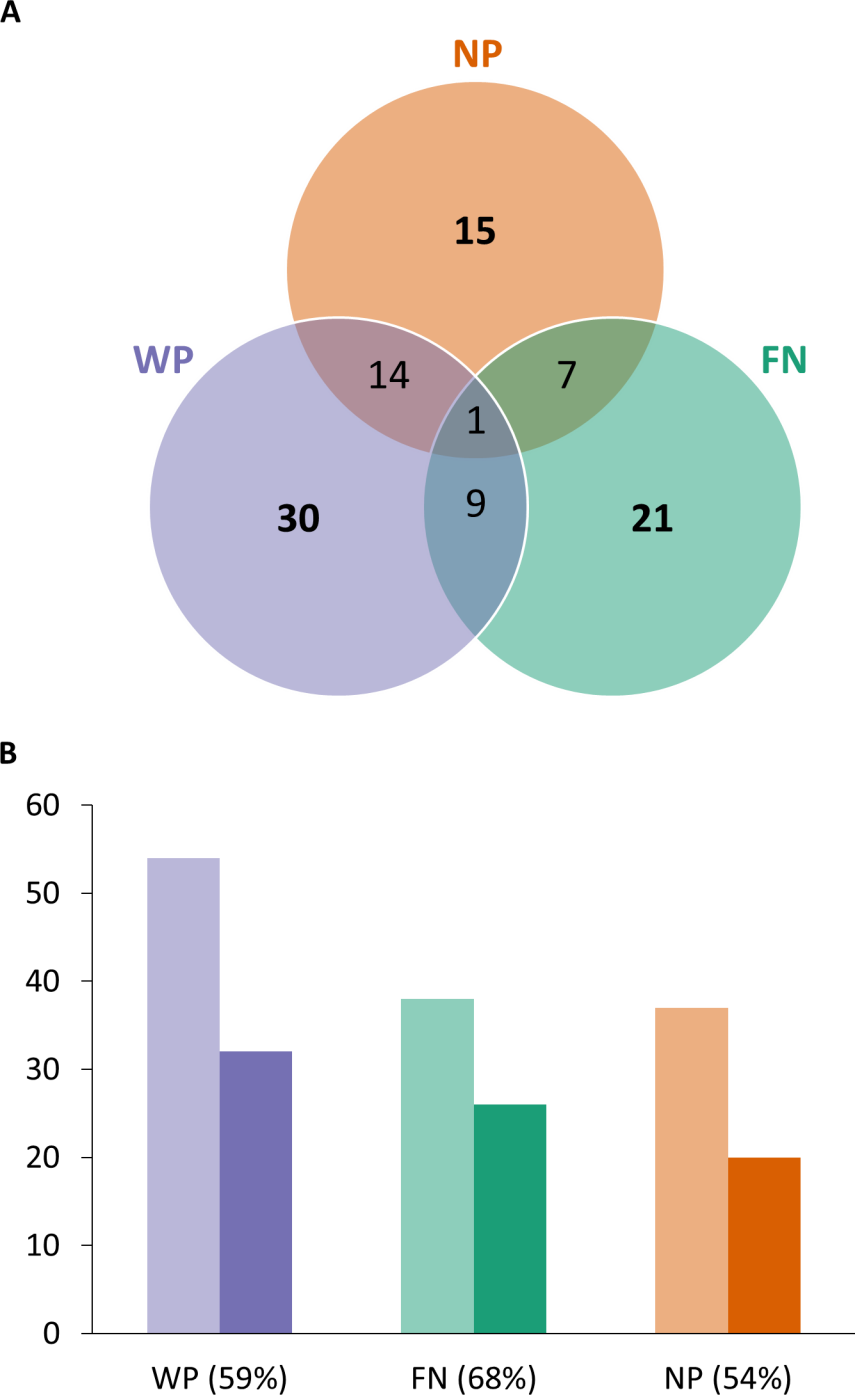
Signatures of selection in the Tasmanian devil genome. Ninety-seven signatures of selection were identified in the Tasmanian devil (*Sarcophilus harrisii*). Their distribution among the three investigated populations is provided in the form of **(A)** a Venn diagram and **(B)** bar plots. Light bars show the total number of signatures of selection identified in each population, among which dark bars (and the proportions between brackets) represent those standing at less than 100 kb from a protein-coding gene having a human orthologue according to Ensembl 90. Populations: FN = Freycinet; NP = Narawntapu; WP = West Pencil Pine.

Our analysis resulted in the identification of 97 signatures of selection dispersed throughout the chromosomes, accounting for about 0.3% of the genome. Most signatures of selection were found to be population-specific, and only one was common to the three investigated populations (Fig 1A). The West Pencil Pine (WP), Freycinet (FN) and Narawntapu (NP) populations displayed 54, 38 and 37 signatures of selection, respectively (Fig 1B). The ability to detect signatures of selection from the dataset was influenced by the experimental sampling protocol and SNP detection, which was specific to each population (Table 1). In the WP population, characterized by an adequate time-series sampling but with a low SNP density, a large number of small signatures of selection were detected. In the NP population, despite a high SNP density, a low proportion of candidate SNP (0.4%) was detected. This can be explained by the non-optimal sampling time-series (Table 1), with the latest data point too close to the date of arrival of the disease, which left little time for selection to produce visible effects. The FN population presented a much better SNP density and sampling time-series, compared to the other populations, which allowed detecting the highest number of candidate SNP (210) and larger signatures of selection. As a consequence, a larger proportion (68%) of candidate regions were located less than 100 kb from a protein coding gene in the FN population (Fig 1B). In total, for the three populations, 60 signatures of selection were detected in the vicinity of protein coding genes with a human orthologue (see S1 Fig and S1 Table for the details) allowing us to identify 148 candidate genes according to Ensembl genome browser 90 (S1 Table).

**Table 1:** Genomic time-series investigated to identify signatures of selection in the Tasmanian devil (*Sarcophilus harrisii*).

Population symbol	Sampling location	DFTD arrival	*N*_*e*_	*N*_*1*_ (*t*_*1*_)^a^	*N*_*2*_ (*t*_*2*_)^a^	*N*_*3*_ (*t*_*3*_)^a^	Nb. of analyzed SNPs	Nb. of candidate SNPs	Prop. of candidate SNPs (%)
FN	Freycinet	2001	34	29 (1999)	-	20 (2012–2013)	16978	210	1.2
NP	Narawntapu	2007	37	26 (1999)	26 (2004)	27 (2009)	27173	104	0.4
WP	West Pencil Pine	2006	26	21 (2006)	-	43 (2013–2014)	5401	88	1.6

*N*_*e*_, effective population size

^a^ Time-series include either two (for FN and WP) or three (for NP) temporal samples. *N*_*i*_ denotes the size of each temporal sample *i* indexed in chronological order. The year of sampling, *t*_*i*_, is provided between brackets.

N.B. Data were made publicly available in [13]. More information about the dataset can be found in [14].

### The functional annotation of candidate genes reveals a strong link with cancer

**Fig 2:**
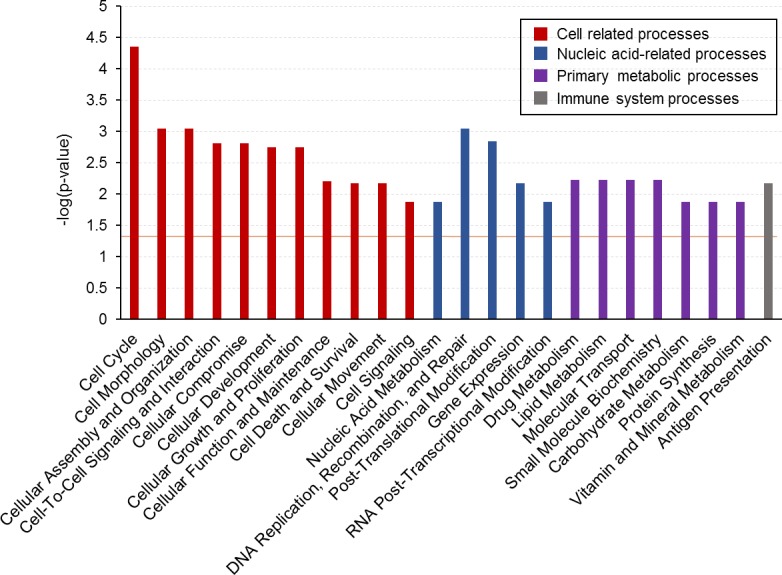
Molecular and functional categories associated with protein coding candidate genes. Representation of overrepresented molecular and functional categories obtained by functional analysis in IPA. Related functional categories are represented with the same color code. Only functions with a -log_10_(p-value) ≥ 1.3 (orange line in the graph, corresponding to a p-value ≤ 0.05) were considered.

We used the IPA knowledge base analysis tool (Ingenuity Systems®, www.ingenuity.com) to identify biological functions and disease-related categories associated with our candidate genes. The top molecular and cellular functions included several “cell-related functions” such as cell cycle, morphology and organization. Other functions were related to nucleic acids, primary metabolism and the immune system, as shown in Fig 2. Among the top 100 disease-related categories, 73 were associated with “cancer” (S2 Table). The “solid tumor” category was the most overrepresented (p-value = 1.16×10^−10^_,_ FDR = 4.39×10^−7^) with 138 genes out of 147 associated with this term. According to IPA, all the 60 signatures of selection were located in the vicinity of coding sequence host genes potentially related to cancer.

Some candidate genes are key regulators of signaling pathways mediating cancer progression involved in cell cycle, apoptosis and genome instability. Among these candidates are the DTWD1, NEK6 and NSD3 genes that are regulators of the G2/M transition [16–18], and BAG4 and TRIM66, which prevent apoptosis [19,20]. The CEP131 and PINX1 genes are involved in chromosome stability [21,22].

A great number of candidate genes are associated with signaling pathways that usually mediate embryonic development but may also have a role in cancer progression. Alterations of the FGFR1 gene, a tyrosine-kinase receptor (RTK), have been described in many tumors [23,24]. Other candidate genes are known to be involved in oncogenic RTK pathways, such as SHC4 [25] and ST5/DENND2B [26]. The CEP131 gene is involved in cell proliferation and migration through the activation of the phosphoinositide 3-kinase (PI3K/Akt) signaling [27] and the SMAD3 gene is a key mediator of the transforming growth factor-β (TGF-β) signaling pathway involved in cancer progression [28]. The candidate FOXN3 is a key gene of the Wnt/β-Catenin pathway that is altered in cancers [29]. Other candidates, such as the SPTBN1, NSD3 and CDK14 genes, are able to influence cancer progression through Wnt signaling [18,30,31]. Key mediators of other pathways related to both development and cancer, such as Notch and Hippo, were among our candidates. The NOTCH2 gene is involved in the direct cell-cell interactions of the Notch pathway that can inhibit cancer progression [32]. The TEAD4 gene belongs to the TEAD (transcriptional enhancer factor domain) transcription factors that are required for activating the proliferation genes targeted by Hippo signaling in cancer [33].

### An important subset of candidate genes are mediators of metastasis

It is noteworthy that a great number of candidate genes identified in the present study have the potential to influence invasiveness and metastasis of cancer cells. In particular, we identified seven metastasis-related genes that encode adhesion molecules (ADGRA2, ADGRD2, CDH8, MCAM, THY1, TSPAN9, and TSPAN11). Other candidates, such as PRRX2 and CST3, confer metastatic properties to cancer cells through the TGF-β pathway [34,35]. Similarly, FOXN3 [36], HMGCS2 [37], LSM1 [38], PHGDH [39], RIN2 [40], TRPM8[41], are known regulators of metastatic processes. In total, over 30 selection candidates identified in this study are known to participate in metastasis-related mechanisms.

### Behavior-related genes among the identified candidate genes

The second major functional category associated with our candidate genes refers to the architecture of complex behaviors. Functions associated with development and the nervous system (S2 Table) were revealed by IPA analysis. In particular, 16 candidates were classed within the annotation term “development of neurons” (S2 Fig). Several of them may contribute to the development and homeostasis of important cellular compartments in the central nervous system (CNS). For example, SYNDIG1 is essential for the formation of excitatory synapses in the hippocampus [42]. NKX2-2 is a key regulator of serotonergic neuron development [43]. SDK2 is an adhesion molecule that regulates synaptic connections, thereby influencing the arrangement of neural circuits in the CNS [44]. KIF13B, a member of the kinesin motor protein superfamily, has a key role in the regulation of axon development [45]. Other candidates have putative roles at the synapse level. SLITRK5 and SLC38A10 play important roles in neurotransmission [46,47]. CDC42EP4 and HERC1 are involved in synapse homeostasis [48,49]. In addition, the candidate list harbors several genes encoding subunits of ion channels (e.g., CACNB2, KCNA4, KCNIP3, or KCTD3) that are involved in signal transmission in the CNS.

Some human homologues of our candidate genes are also associated with behavioral disorders. For example, the large signature of selection displayed on scaffold GL834709 (nucleotides 2,702,597 to 2,946,330) corresponds to human locus 8p11.23, which is related to cancer [50], but has also been proposed as a “neurodevelopmental hub” associated with autism spectrum disorders (ASD) according to a recent meta-analysis [51]. The NDUFAF5 candidate is involved in the assembly of the first complex of the respiratory chain [52], which is frequently impaired in CNS disorders like ASD [53]. Several other candidates, such as CACNB2 [54], CDH8 [55], HERC1 [56], KCTD3 [57], KIF13B [58], SERINC2 [59], and SLC39A11 [60] have been associated with ASD. Some candidates have been linked to intellectual disability (ID), such as CRBN [61], GPKOW [62], HERC1 [56] and KCNA4 [63], or to other behavioral disorders, such as CDC42EP4 [64] and SLITRK5 [46].

### A few candidates may contribute to immunity

Immunity, a function that may be related to cancer progression, is also represented through a few genes. In particular, six candidates (TRIM10, TRIM15, TRIM26, TRIM39-RPP21, TRIM62, and TRIM66) are members of the large tripartite motif (TRIM) family that consists of ubiquitin ligases involved in both innate immunity and cancer progression [65]. With the exception of TRIM39-RPP21, all the TRIM genes identified here are known to be related to cancer according to IPA (S1 Table). TRIM10, TRIM15, TRIM26, and TRIM39-RPP21 belong to the human leukocyte antigen (HLA) region, which gathers many immunity regulators [66]. TRIM62 and TRIM66 have also been suggested to have a role in immunity [67,68]. Candidates from the TSPAN (TSPAN9, TSPAN11) and the aGPCR (ADGRA2, ADGRD2) families, which were cited above for their putative roles in metastasis, may also be implicated in immune response [69,70]. The versatile SMAD3 candidate has been shown to influence the immunosurveillance of cancer [71].

## Discussion

The Tasmanian devil dataset investigated here was initially presented and analyzed by Epstein et al. [14], who reported two putatively selected regions harboring seven candidate genes. These two regions were also detected in our analysis and correspond to signatures of selection located in scaffolds GL841593 (nucleotides 4,501,785 to 4,979,756) and GL849657 (nucleotides 283,671 to 283,701). Epstein et al. [14] performed their analysis with a composite test statistic that took into account temporal changes in both allele frequency and integrated extended haplotype homozygosity of an individual SNP site (iES) [72]. Such an approach is based on the idea that an SNP undergoing strong selection would rapidly rise in frequency, while the haplotypic diversity of the surrounding region would suddenly decrease. To avoid false-positives, Epstein et al. [14] restricted their analysis to the candidate regions shared by the three populations. The limit of this approach is that the total number of candidate genes detectable is highly dependent on the SNP density, especially since the West Pencil Pine (WP) population presented a low genotyping density with only ~ 5000 available SNP (Table 1).

We chose a different approach than that proposed in [14]. First, the three devil populations were analyzed separately since there is substantial genotyping heterogeneity among populations (Table 1). In addition, the genetic structuration of the Tasmanian devil implies that some observed polymorphisms are expected to be population-specific [10,12]. In particular, the existence of population-specific genotypes has already been emphasized to account for some phenotypic differences in response to DFTD among devil populations [73]. Second, we used a customized method that looks for selection in each population independently. Our method is based on a Wright-Fisher model coupled to maximum-likelihood computations, which makes it possible to determine whether the observed temporal variation in SNP frequency is more likely to be caused by selection and drift than by drift alone in each population. Importantly, our method implements a test statistic that takes into account the amount of drift through the effective population size (*N*_*e*_), in each population. Despite the very low *N*_*e*_ in the Tasmanian devil (*N*_*e*_ ~ 30, see [14]), our method allowed the identification of 97 signatures of selection (Fig 1A). This confirms that the selection imposed to the Tasmanian devil during the emergence of DFTD was extremely intense and that the evolutionary response to a transmissible cancer may involve the contribution of many genes, which is in line with the complex multistep processes of cancer [74].

Our analysis identified a majority of population-specific candidate regions for DFTD-linked selection (Fig 1A). The low number of candidate region overlapping among populations may be explained by different factors. The first relates to the large sampling heterogeneity in genotyping and in SNP densities among populations, as demonstrated by the fact that only 5% of the SNP were common among populations. The second reason involves the complex host-cancer interaction, which strongly depends on the genetic variation available in the host population. We can expect selection to act on different genes in different populations because between-population genetic variation exists. This does not necessarily imply that the adaptive mechanisms selected to resist cancer are dramatically different among devil populations; rather, this refers to the redundancy of gene functions, with different genes or different pathways being able to act in a similar and functional manner [75].

The Tasmanian devil-DFTD interaction is a nice example of host-pathogen reciprocal evolutionary process. DFTD-driven selection acts to increase host resistance and reduce the negative effects of the tumor on individual fitness. In an evolutionary antagonistic process, DFTD evolves to counter-adapt in response to the host adaptive changes [76]. Our study, as in the previous analysis by Epstein et al. [14], focuses on the genetic changes arising in the host, rather than the tumor itself.

DFTD-driven selection has targeted genes in the host cells of the tumor microenvironment [77,78], where non-cancerous cells such as fibroblasts, adipocytes, inflammatory cells, etc., contribute to the malignant progression [79]. Genes selected in the host may therefore limit normal cell recruitment and activation by the cancerous cells [80], and regulate metastasis-related processes [81,82].

Through our analysis, we identified more than 30 candidate genes that may contribute to metastasis. In particular, some of them belong to different families of cell adhesion molecules (e.g., tetraspanins, cadherins, adhesion G protein-coupled receptors, immunoglobulins), which are essential in processes that lead to metastasis [83]. For example, both MCAM and THY1, which encode cell adhesion molecules of the immunoglobulin superfamily (IgSF-CAMs), are frequently overexpressed in metastatic tumor tissues [84,85]. Moreover, tetraspanins and adhesion G protein-coupled receptors also influence metastasis [86–88] as well as the cadherin CDH8 [89]. These results must be considered in the light of the high prevalence of DFTD metastases in infected devils [7] and the importance of cell adhesion processes in shaping the tumor microenvironment [82]. All this suggests that the control of metastasis, in particular through the maintenance of the host tissue integrity, could be an essential component of the host evolutionary response to DFTD.

In addition to the strong association with metastasis mentioned above, the functional annotation of our candidate genes allowed identifying putative key mediators of cellular processes frequently deregulated in cancers, such as cell cycle control and apoptosis. Well-known signaling pathways related to both cancer and development, such as MAP-kinase, TGF-β and Wnt pathways, were also targeted by selection. In a marsupial such as the Tasmanian devil, the development of neonates represents a critical window for selection [90]. As a consequence, we must consider the possibility that selection has targeted some genes for their developmental role or even for their specific tumor-suppressive action during development [90].

Overall, our analysis suggests that natural selection may have targeted multiple cellular circuits to limit the acquisition of cancerous features in devil tumor tissues [74,79], which should ultimately prevent or at least delay cancer growth and metastatic processes. In the context of a transmissible cancer, such genetic changes are expected to provide the host with increased fitness, since host individuals with a slow DFTD progression will be able to reproduce despite infection [9,91].

Among the target genes identified in this study, there are several human orthologues associated with behavioral disorders affecting synaptic connections and characterized by deficits in communication and social interaction, such as ASD and ID [92–94]. This observation is very intriguing, especially in the light of recent results indicating that more aggressive individuals were at greater risk of developing DFTD [11,91,95]. DFTD-driven selection could therefore have favored less aggressive individuals, which are less likely to be infected due to reduced physical interactions with other Tasmanian devils [91]. This empirically supports the hypothesis recently discussed in [2] that an evolutionary response to cancer could rely not only on cellular pathways involved in cancer progression, but also on adjustments of life-history traits and behavior. Even if evolutionary costs driven by sexual selection may be opposed–because socially dominant individuals engage more often in mating [91]–, Roche et al. [2] suggest that “the avoidance of contagious cancers could be a selective force for specific behavior”, which agrees with our results. The Tasmanian devil-DFTD interaction provides a good natural model for further investigating this hypothesis through additional field and resequencing studies.

Overall, our results suggest that DFTD has extensively shaped the genome of the Tasmanian devil and that several functions have been targeted in the host by DFTD-driven selection. This supports the evidence that adaptation occurs rapidly even in situations of limited genetic variation. Our study also shows that genomic time-series data are particularly useful for detecting signatures of selection associated with complex phenotypes even when the number of generations of selection investigated (~ 5 generations) and the effective population size (*N*_*e*_ ~ 30) are very small. In this study, we only had the opportunity to explore the host perspective of the host-tumor interaction. Further studies incorporating genomic time-series data from both the host and the transmissible tumor will be required to better understand the host-tumor coevolution.

## Methods

### Tasmanian devil dataset

The data analyzed in the present article were initially reported in [14] and made publicly available as a Dryad data package [13]. This data package consists of SNP genotyping data produced by Stacks [96] following RAD-seq (Restriction-site Associated DNA sequencing) assays [97] from tissue samples collected in 360 Tasmanian devils across Tasmania. Samples were collected at different time points between 1999 and 2014, allowing the analysis of genomic time-series that take into account the impact of DFTD through time on Tasmanian devil populations. We restricted our analysis to the same samples as in [14], from the localities of Freycinet (FN), Narawntapu (NP) and West Pencil Pine (WP). We reproduced the SNP filtering strategy reported in [14]. In brief, we performed filtering according to (i) MAF computed over the whole dataset (SNP with MAF less than 0.01 were discarded), (ii) observed heterozygosity computed over the whole dataset (SNP with heterozygosity over 0.5 were discarded), (iii) the proportion of missing genotypes (SNP with less than one-third of genotypes either in the whole dataset or in a sample were discarded, except for the two smallest samples in which SNP with less than half genotypes were removed), (iv) the linkage disequilibrium between neighboring SNP (using PLINK [98], we removed one SNP from pairs of SNP harboring *R*^*2*^> 0.99 over 20 successive SNP and 50 kb of distance in any sample). We obtained filtered datasets of 16978, 27173 and 5401 SNP for the FN, NP and WP populations, respectively. Relevant information about the dataset for the present work is summarized in Table 1. Further information about sample collection, genotyping and data processing can be found in [14].

### Signatures of selection

We submitted the genomic time-series of the three investigated Tasmanian devil populations to a customized method for detecting footprints of selection (available at https://github.com/hubert-pop/signasel). This method was initially described and successfully implemented to detect genomic regions targeted by short-term selection in experimental wheat populations [15]. Briefly, this method compares two Wright-Fisher models, one including drift and the other including drift plus selection, in a maximum-likelihood framework. The model that best fits the SNP frequency variation observed over time is identified through a Likelihood Ratio Test (LRT). Each SNP is individually tested and associated with a p-value that quantifies to what extent the temporal variation in SNP frequency may be due to selection, under a null hypothesis postulating an effect of drift only. A strength of this method is to model and disentangle the effects of drift plus selection as opposed to those of drift alone in a population-specific manner. This has proven to be efficient for detecting SNP under selection from genomic temporal samples separated by a few generations in small populations undergoing intense selection, as in the Tasmanian devil. For example, we performed simulations mimicking a strong selection (applying a constant selection coefficient of 0.5) in the FN population (considering a constant effective population size, *N*_*e*_, of 34, and a sampling interval of 6 generations). In such a case, the power to detect a truly selected SNP was 61%, while the false-positive rate was about 1%, suggesting that our method would provide enough power to find signatures of selection from the Tasmanian devil dataset. In the real data analysis, we corrected for multiple testing by applying the Benjamini-Hochberg false-discovery rate (FDR) procedure [99]. Therefore, we considered as relevant signatures of selection the genomic regions that included at least one SNP with a p-value < 0.0001 (which corresponds to a FDR of ~ 8%) or at least two neighboring SNP with p-values < 0.01 (which corresponds to a FDR of ~ 13.5%). We looked for candidates for selection by applying our method to two temporal samples in the FN (the sample from 1999 and the combination of those from 2012 and 2013) and WP (the sample from 2006 and the combination of those from 2013 and 2014) populations, and to three temporal samples (samples from 1999, 2004 and 2009) in the NP population (Table 1). Given the sampling times and an assumed generation time of 2 years in the Tasmanian devil [14], we considered that the time-series covered a period of 6, 5, and 3 complete generations in the FN, NP, and WP populations, respectively. As our method relies on Wright-Fisher models, the effective size (*N*_*e*_) of each investigated population must be provided. Simulations indicated that a bias in the estimation of *N*_*e*_ may under some circumstances affect the power to detect selection but has almost no impact on the false-positive rate (data not shown). We used the values of *N*_*e*_ suggested in [14], that is, 34, 37 and 26 for the FN, NP and WP populations, respectively. These estimates come from the Jorde and Ryman method [100] for inferring unbiased contemporary *N*_*e*_ from genetic data.

### Candidate gene identification

We identified as candidate genes all the protein coding genes standing at less than 100 kb from the detected signatures of selection and having a human orthologue according to the Ensembl genome browser 90 (https://www.ensembl.org). Ensembl stable ID of candidate genes and corresponding orthologues were retrieved using the Ensembl Genes 90 database and the Devil_ref v7.0 Tasmanian devil reference assembly [101] obtained from BioMart.

### Ingenuity pathway analysis

We used the manually curated database IPA® (Ingenuity Pathway Analysis, QIAGEN Inc., https://www.qiagenbioinformatics.com/products/ingenuity-pathway-analysis) to identify the diseases and developmental disorder as well as the molecular and cellular functions associated with our candidate genes. We submitted the list of 147 human orthologues of our candidate genes to a “Core Analysis” with the “Ingenuity Knowledge Base” reference set to find overrepresented functions and diseases in our gene set. This is achieved by IPA by applying a right-tailed Fisher Exact test to estimate the likelihood that the overlap between the set of genes and a given function or disease is due to random chance.

## Supporting information

S1 Fig Sixty signatures of selection were identified in 53 scaffolds in the Tasmanian devil genome at less than 100 kb from a protein coding gene having a human orthologue. (PDF)

S2 Fig Sixteen candidate genes are associated with the annotation term ‘development of neurons’. (TIFF)

S1 Table List of candidate genes for DFTD-driven selection in the Tasmanian devil. (XLSX)

S2 Table Top hundred annotation terms associated by IPA with the list of candidate genes. (XLSX)
