# Supplementary material for: Cancer- and behavior-related genes are targeted by selection in the Tasmanian devil (Sarcophilus harrisii)
Source: PLoS One. 2018 Aug 13;13(8):e0201838. doi: 10.1371/journal.pone.0201838 (PMC6089428; doi:10.1371/journal.pone.0201838)
Supplement: S1 Fig — (PDF) [file pone.0201838.s001.pdf]

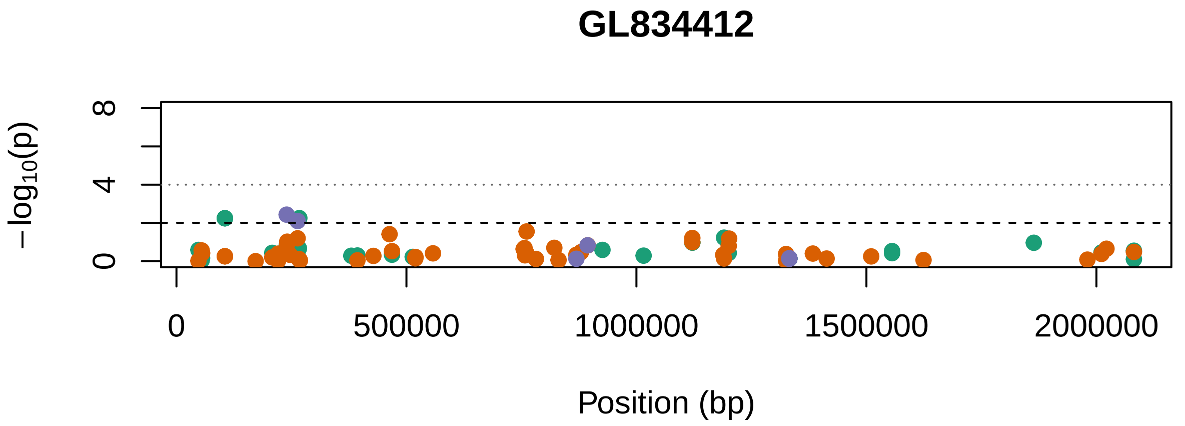
GL834412
−log10(p)
0
4
8
0
500000
1000000
1500000
2000000
Position (bp)

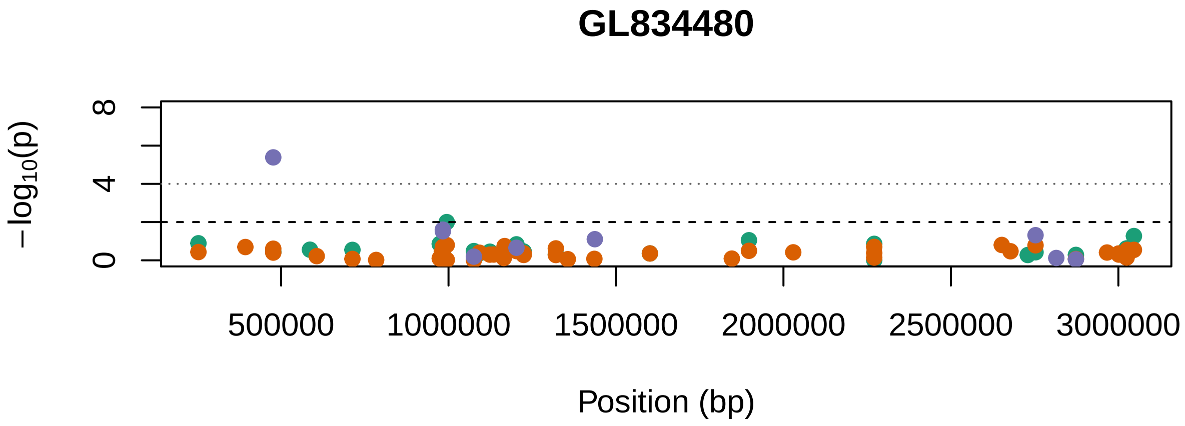
GL834480
−log10(p)
0
4
8
500000
1000000
1500000
2000000
2500000
3000000
Position (bp)

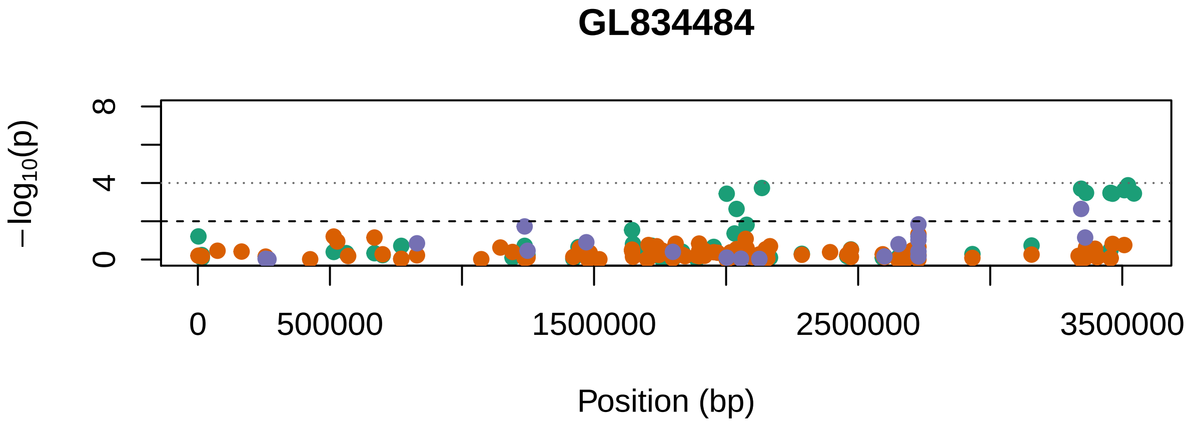
GL834484
−log10(p)
0
4
8
0
500000
1500000
2500000
3500000
Position (bp)

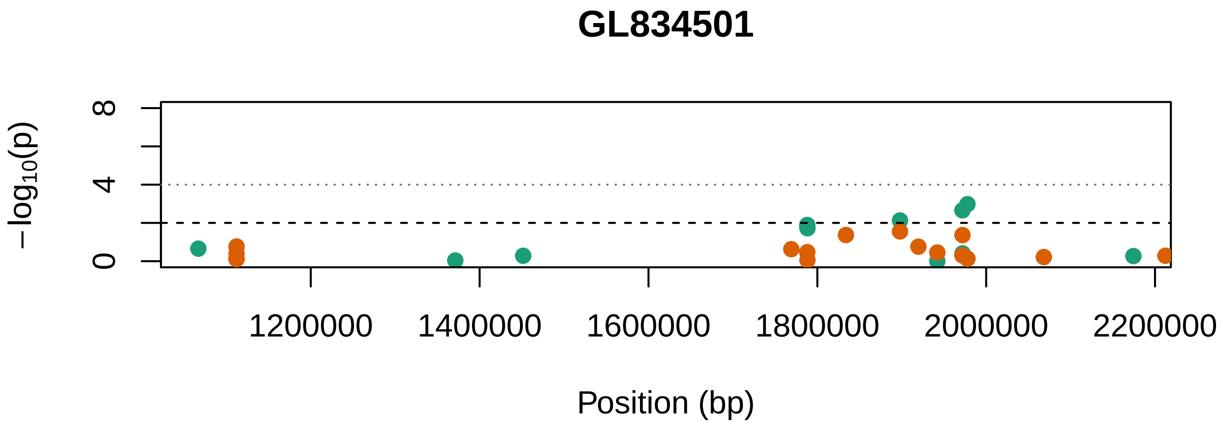
GL834501
−log10(p)
0
4
8
1200000
1400000
1600000
1800000
2000000
2200000
Position (bp)

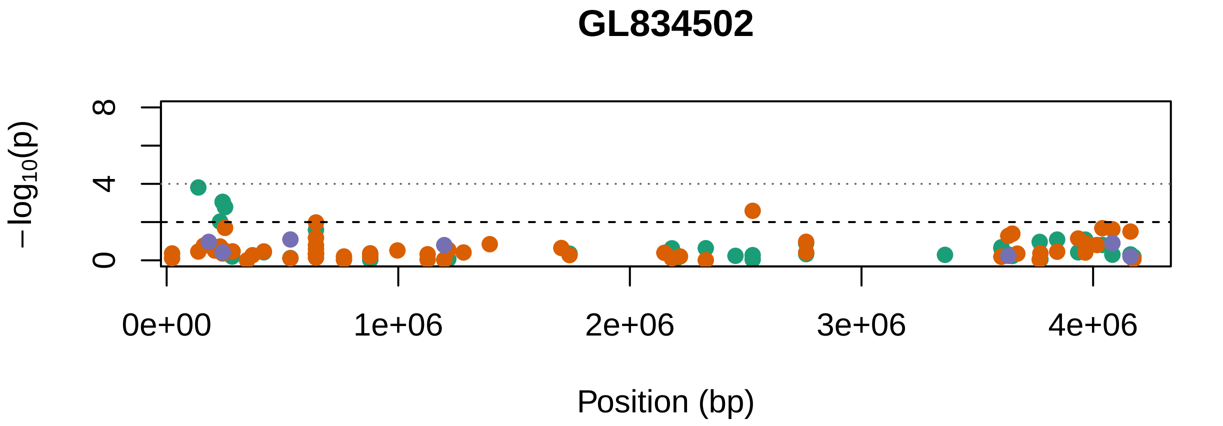
GL834502
−log10(p)
0
4
8
0e+00
1e+06
2e+06
3e+06
4e+06
Position (bp)

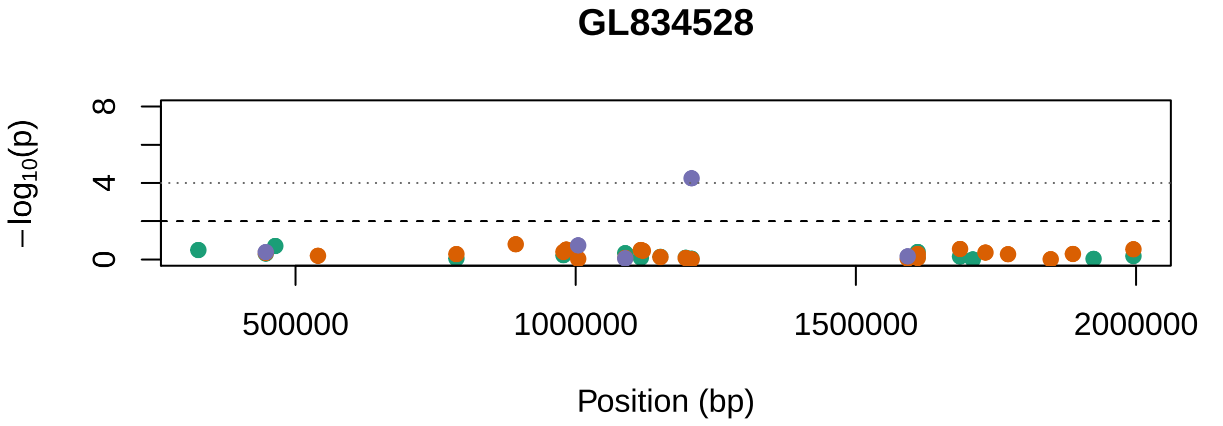
GL834528
−log10(p)
0
4
8
500000
1000000
1500000
2000000
Position (bp)

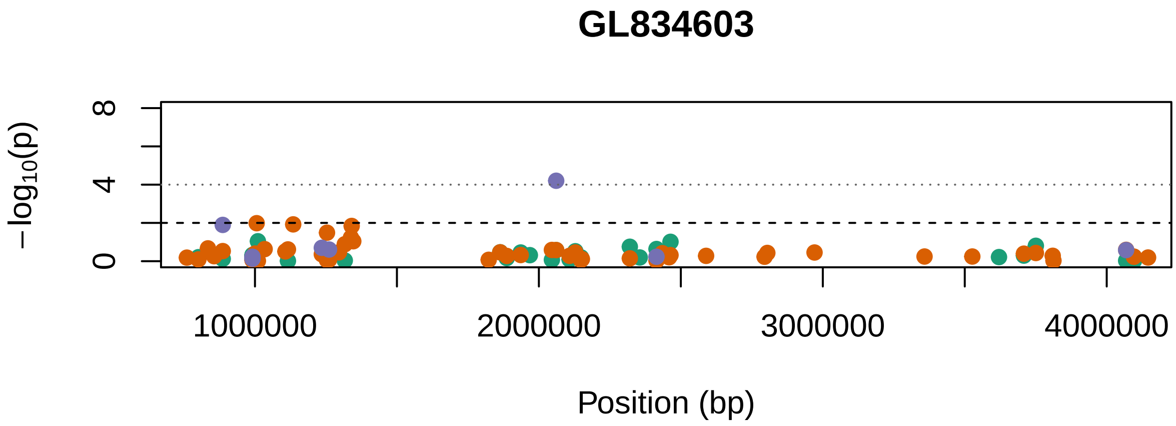
GL834603
−log10(p)
0
4
8
1000000
2000000
3000000
4000000
Position (bp)

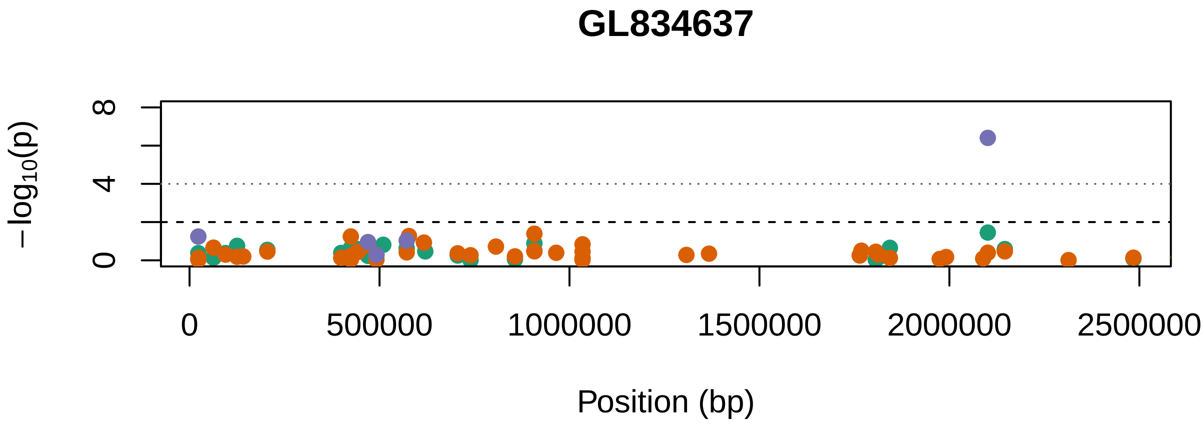
GL834637
−log10(p)
0
4
8
0
500000
1000000
1500000
2000000
2500000
Position (bp)

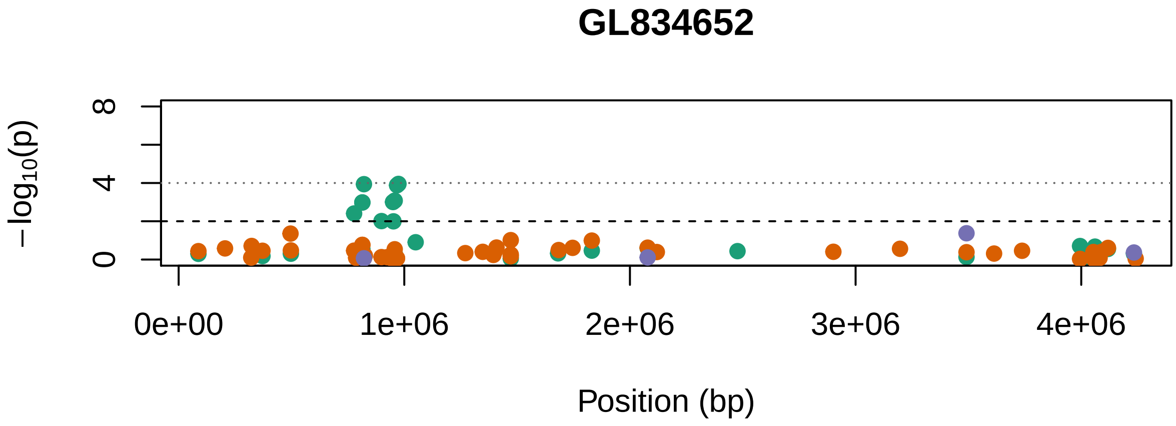
GL834652
−log10(p)
0
4
8
0e+00
1e+06
2e+06
3e+06
4e+06
Position (bp)

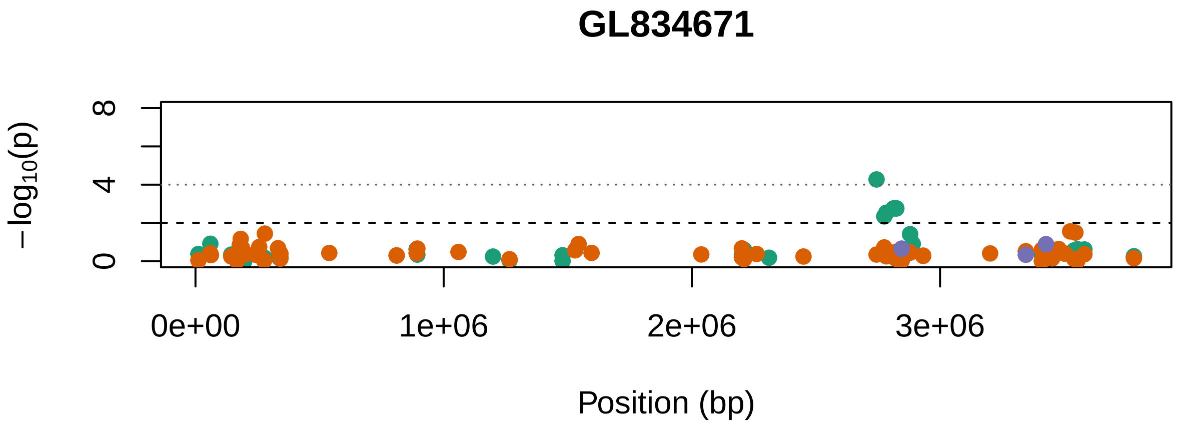
GL834671
−log10(p)
0
4
8
0e+00
1e+06
2e+06
3e+06
Position (bp)

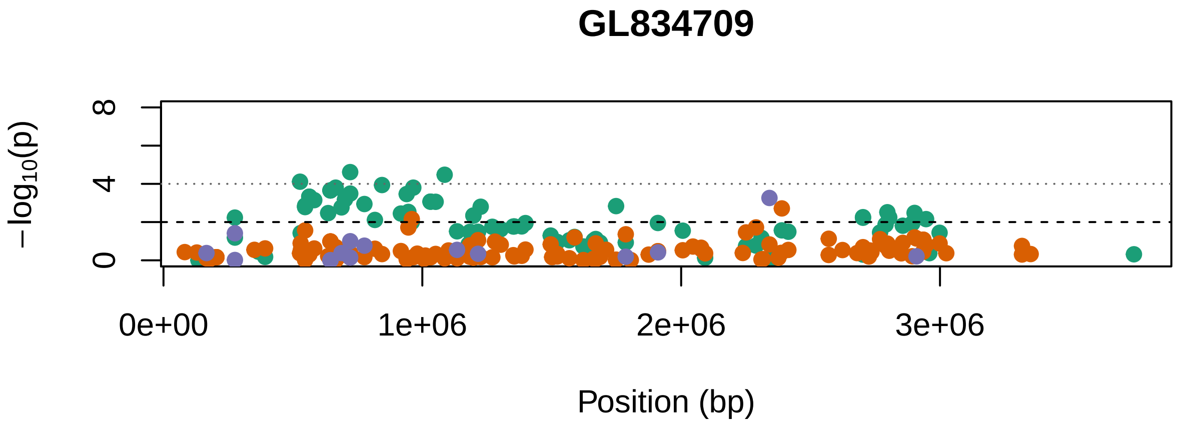
GL834709
−log10(p)
0
4
8
0e+00
1e+06
2e+06
3e+06
Position (bp)

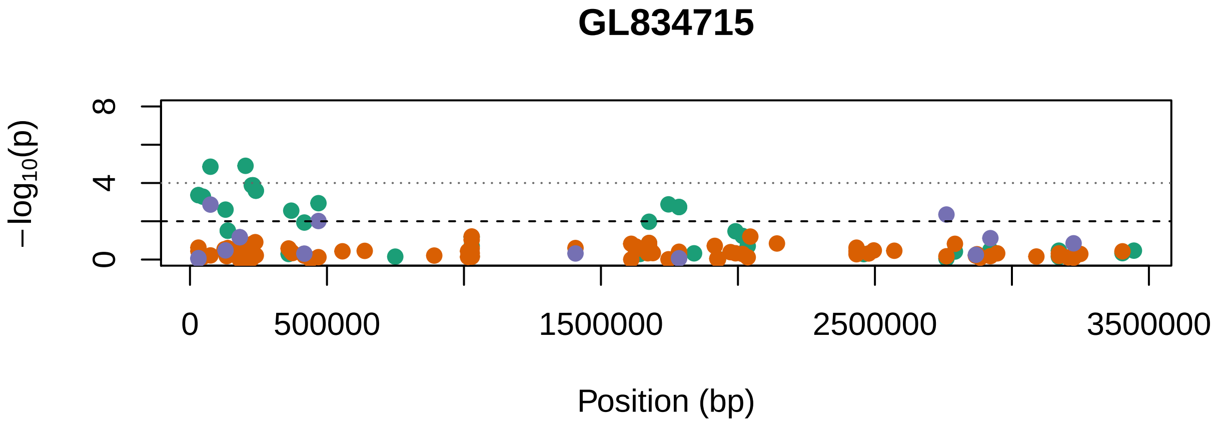
GL834715
−log10(p)
0
4
8
0
500000
1500000
2500000
3500000
Position (bp)

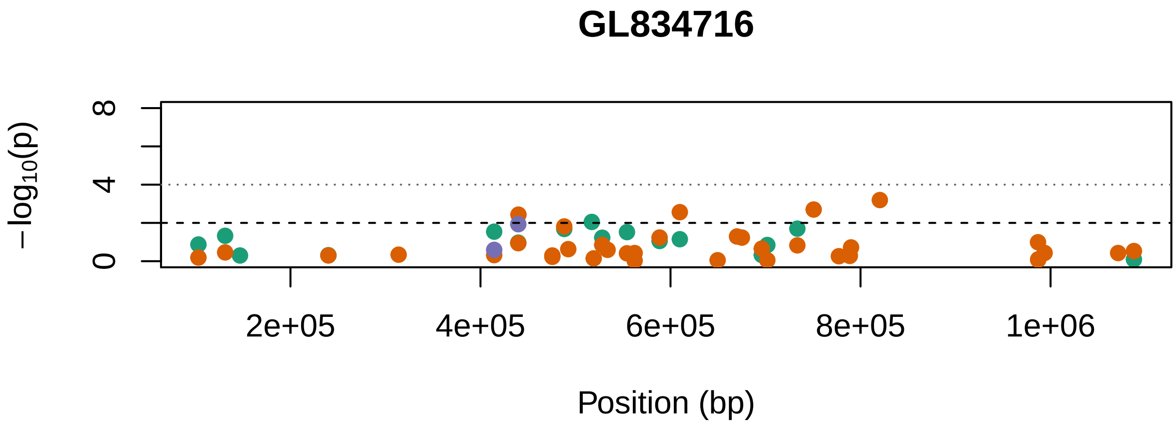
GL834716
−log10(p)
0
4
8
2e+05
4e+05
6e+05
8e+05
1e+06
Position (bp)

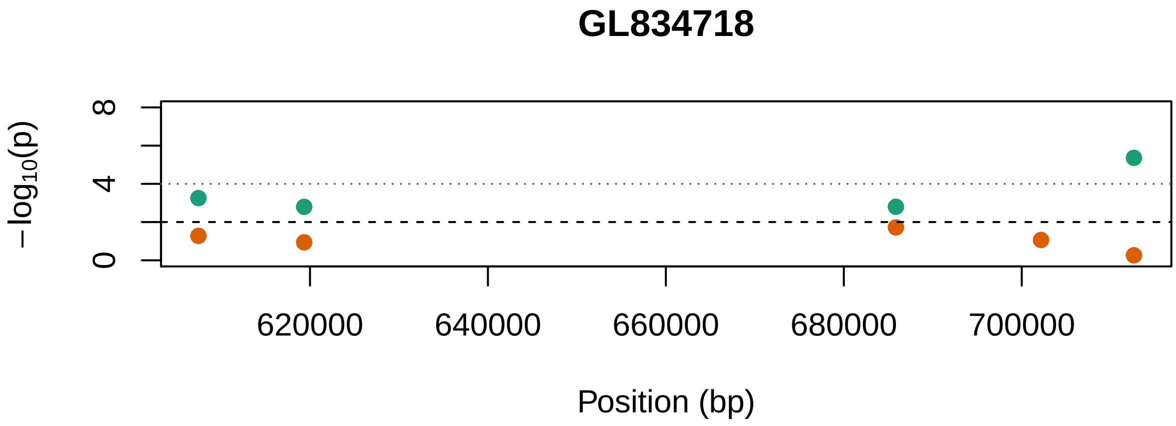
GL834718
−log10(p)
0
4
8
620000
640000
660000
680000
700000
Position (bp)

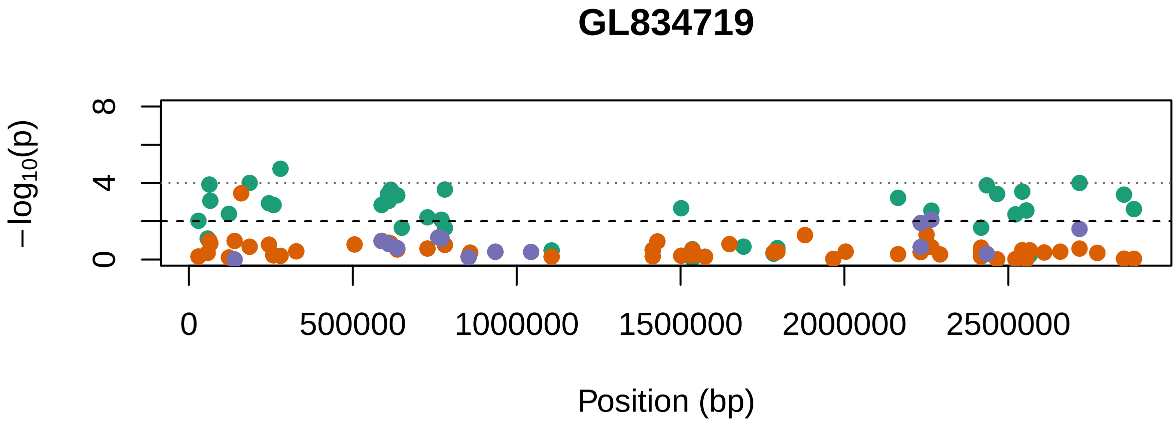
GL834719
−log10(p)
0
4
8
0
500000
1000000
1500000
2000000
2500000
Position (bp)

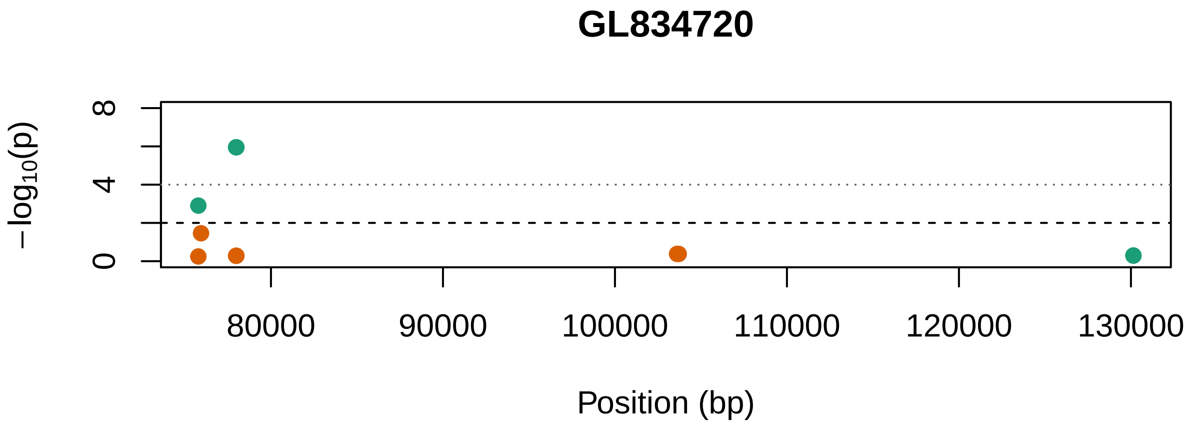
GL834720
−log10(p)
0
4
8
80000
90000
100000
110000
120000
130000
Position (bp)

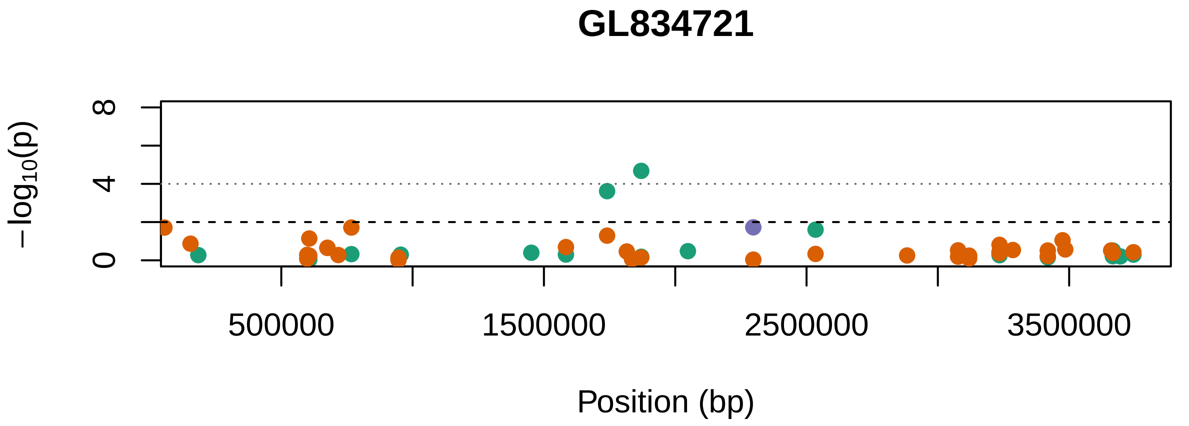
GL834721
−log10(p)
0
4
8
500000
1500000
2500000
3500000
Position (bp)

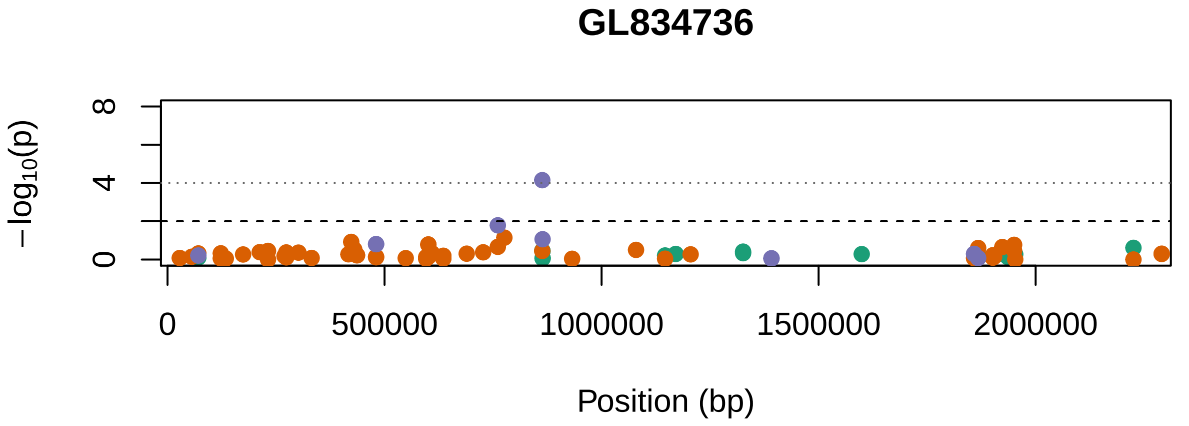
GL834736
−log10(p)
0
4
8
0
500000
1000000
1500000
2000000
Position (bp)

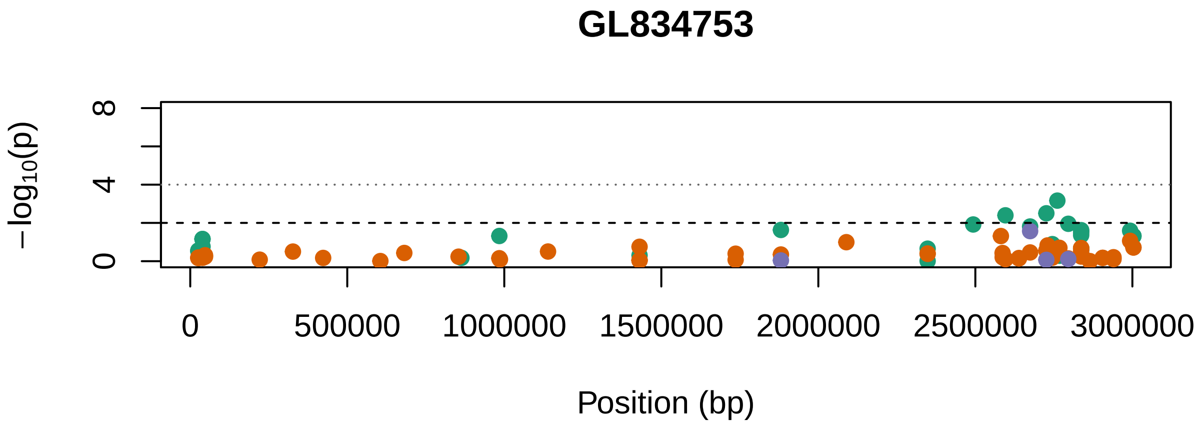
GL834753
−log10(p)
0
4
8
0
500000
1000000
1500000
2000000
2500000
3000000
Position (bp)

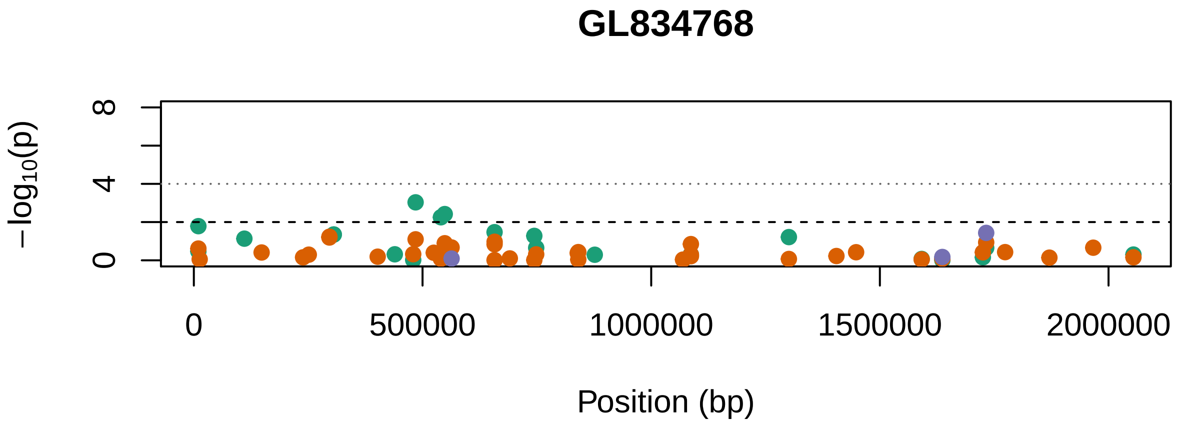
GL834768
−log10(p)
0
4
8
0
500000
1000000
1500000
2000000
Position (bp)

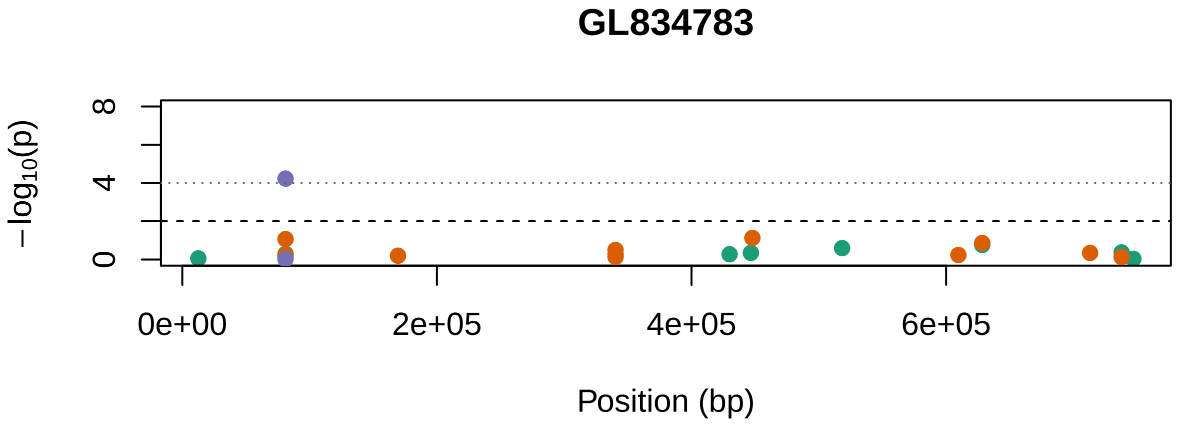
GL834783
−log10(p)
0
4
8
0e+00
2e+05
4e+05
6e+05
Position (bp)

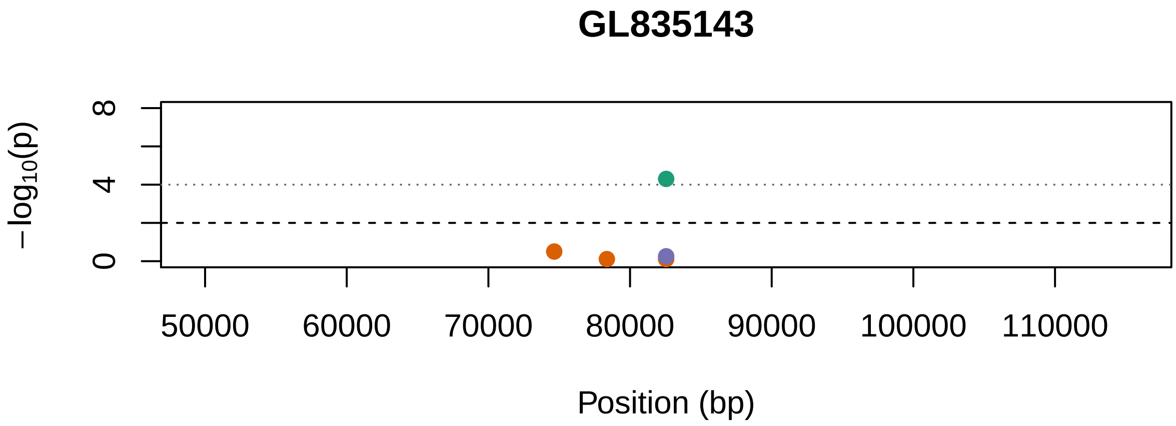
GL835143
−log10(p)
0
4
8
50000
60000
70000
80000
90000
100000
110000
Position (bp)

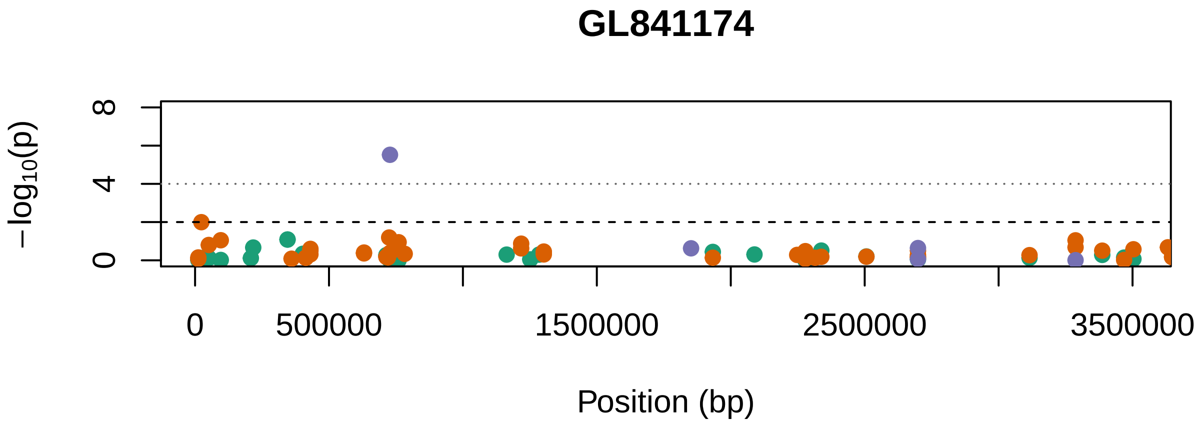
GL841174
−log10(p)
0
4
8
0
500000
1500000
2500000
3500000
Position (bp)

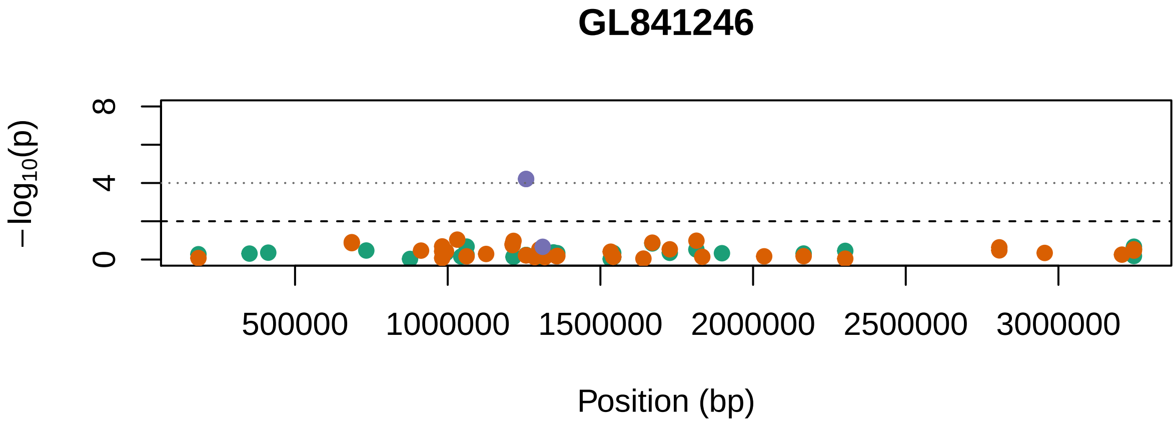
GL841246
−log10(p)
0
4
8
500000
1000000
1500000
2000000
2500000
3000000
Position (bp)

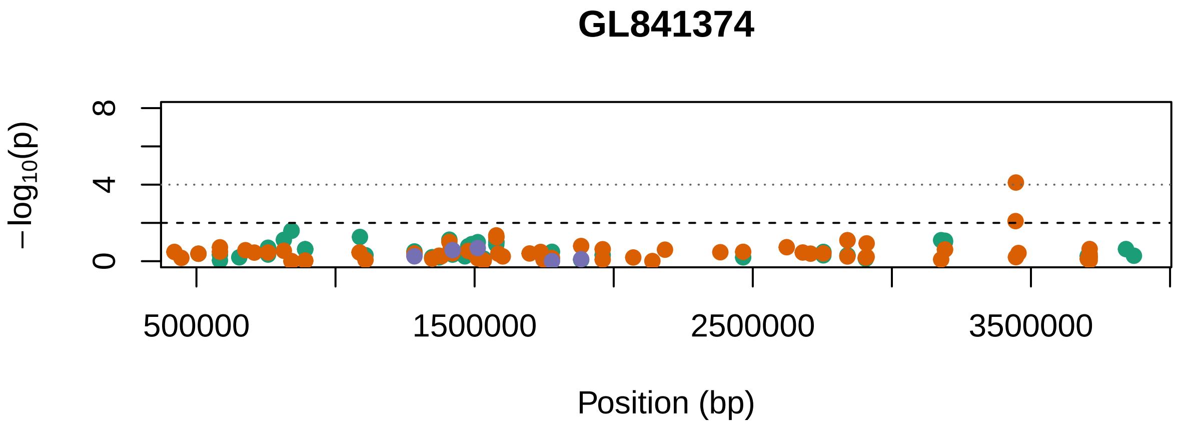
GL841374
−log10(p)
0
4
8
500000
1500000
2500000
3500000
Position (bp)

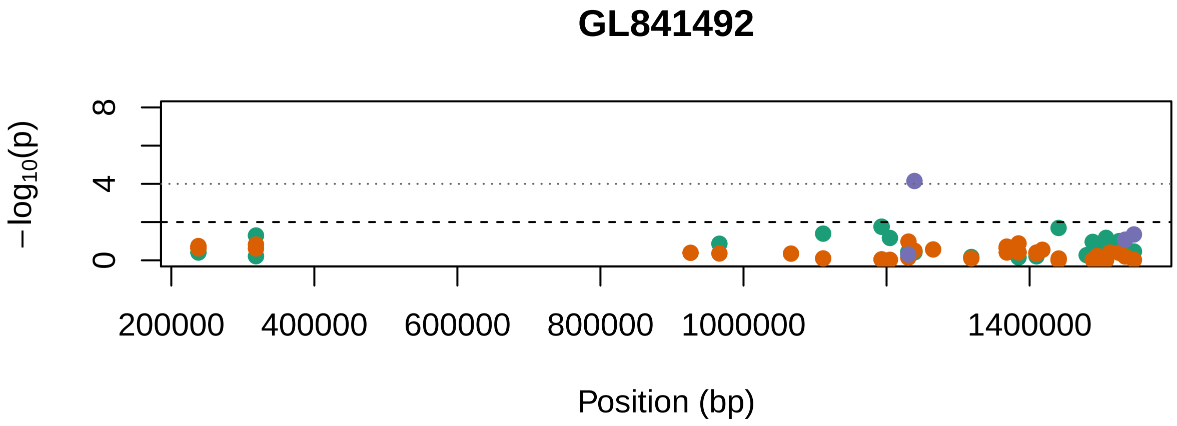
GL841492
−log10(p)
0
4
8
200000
400000
600000
800000
1000000
1400000
Position (bp)

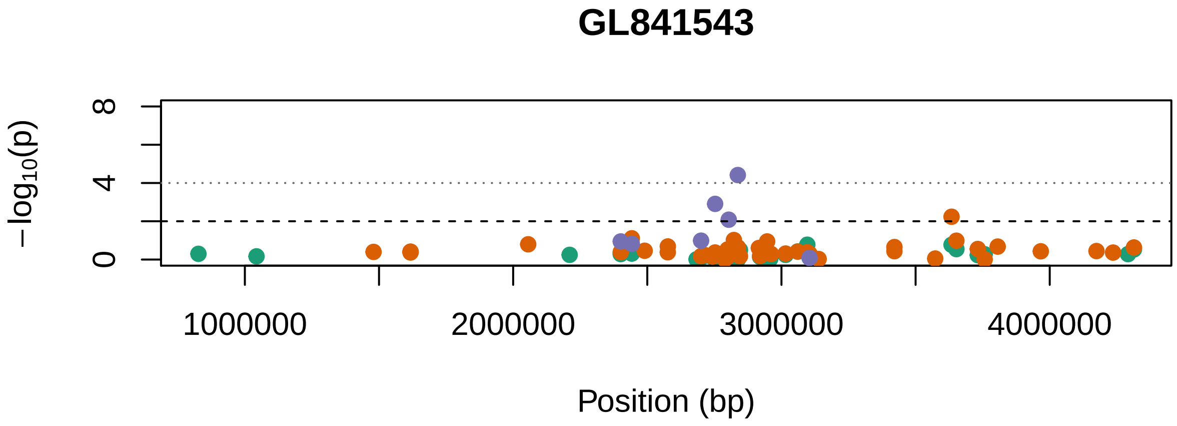
GL841543
−log10(p)
0
4
8
1000000
2000000
3000000
4000000
Position (bp)

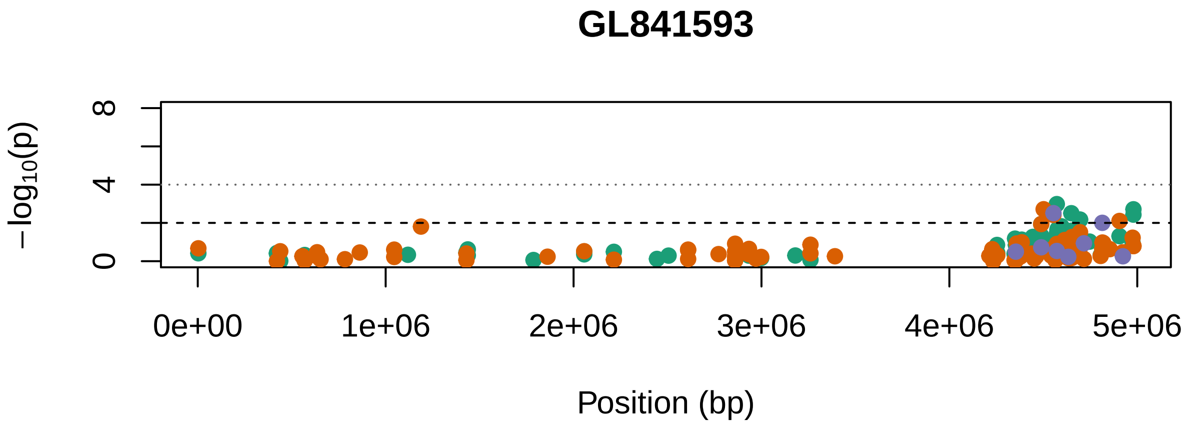
GL841593
−log10(p)
0
4
8
0e+00
1e+06
2e+06
3e+06
4e+06
5e+06
Position (bp)

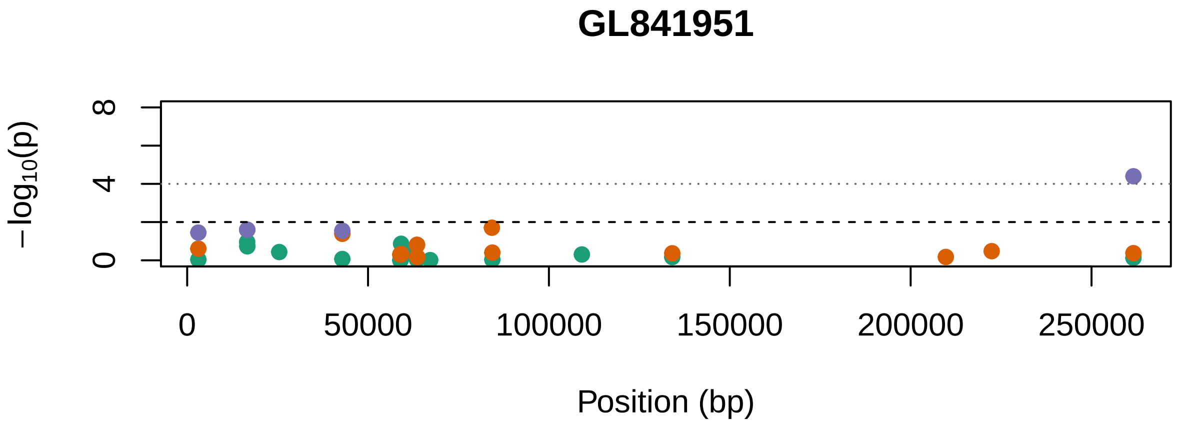
GL841951
−log10(p)
0
4
8
0
50000
100000
150000
200000
250000
Position (bp)

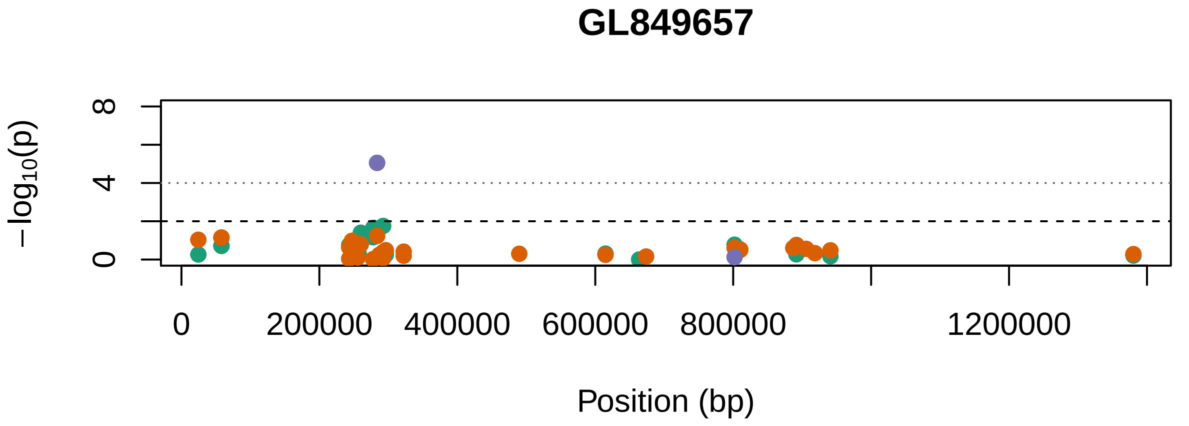
GL849657
−log10(p)
0
4
8
0
200000
400000
600000
800000
1200000
Position (bp)

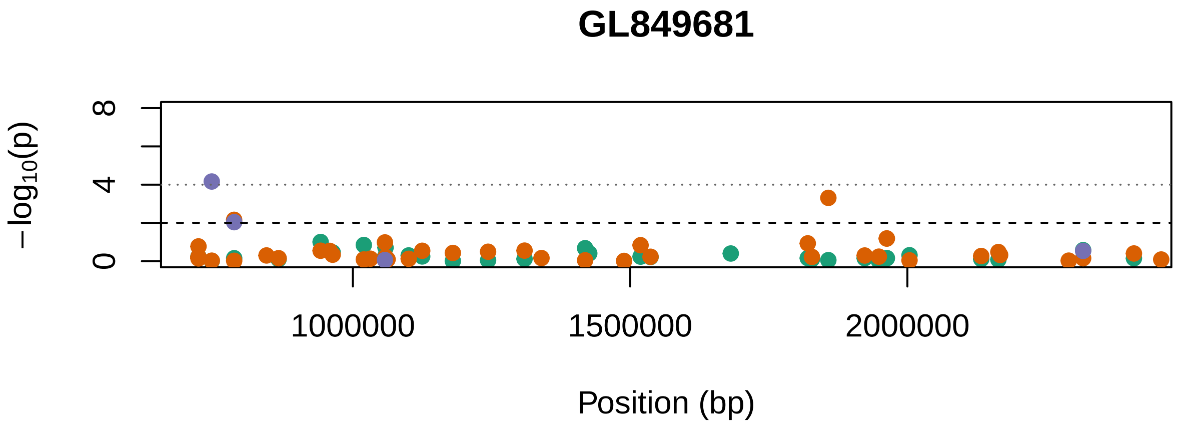
GL849681
−log10(p)
0
4
8
1000000
1500000
2000000
Position (bp)

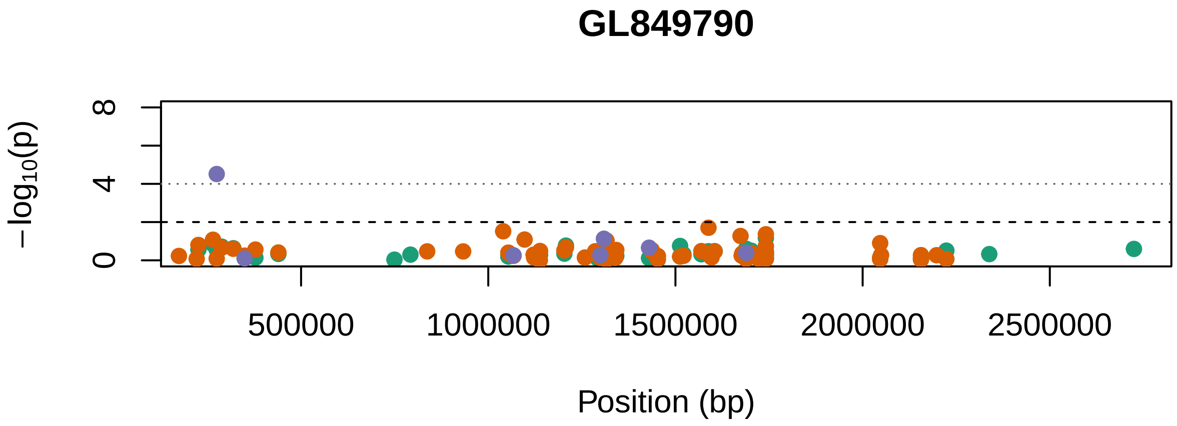
GL849790
−log10(p)
0
4
8
500000
1000000
1500000
2000000
2500000
Position (bp)

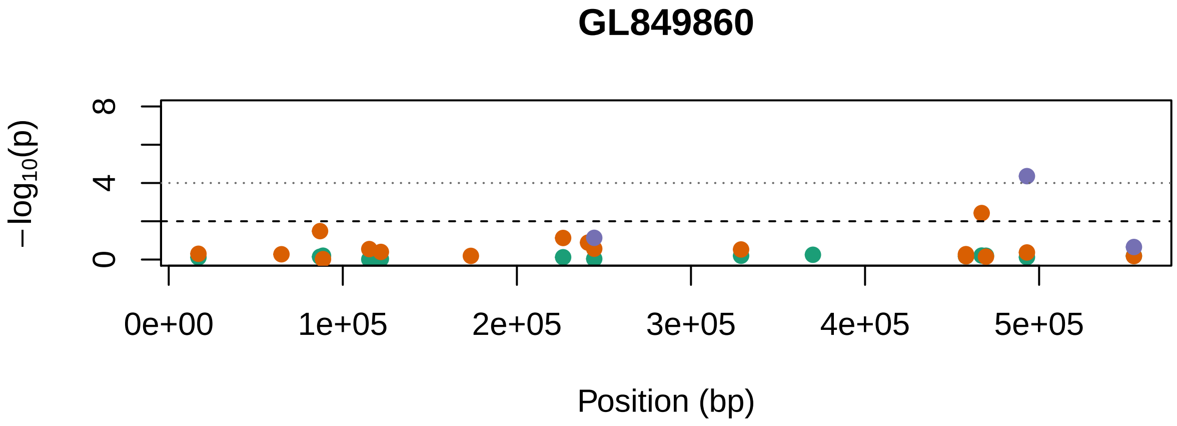
GL849860
−log10(p)
0
4
8
0e+00
1e+05
2e+05
3e+05
4e+05
5e+05
Position (bp)

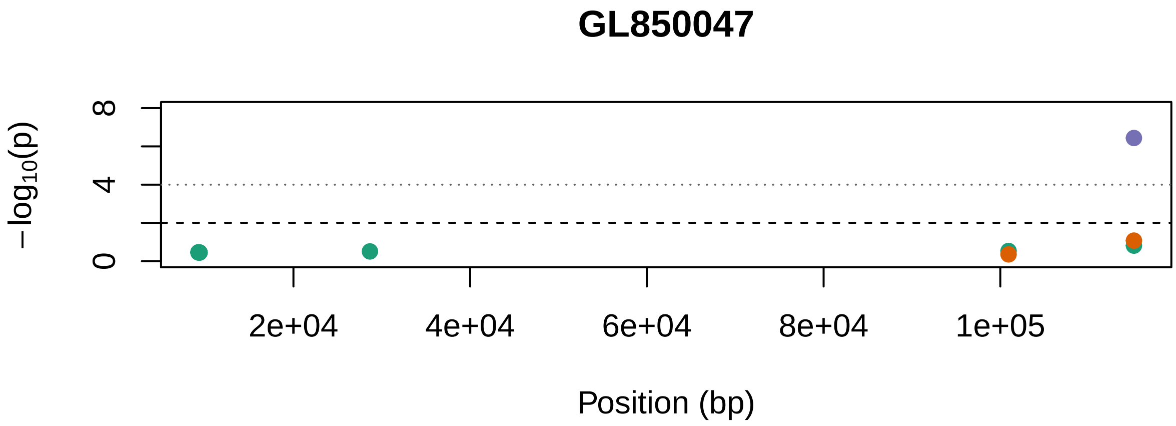
GL850047
−log10(p)
0
4
8
2e+04
4e+04
6e+04
8e+04
1e+05
Position (bp)

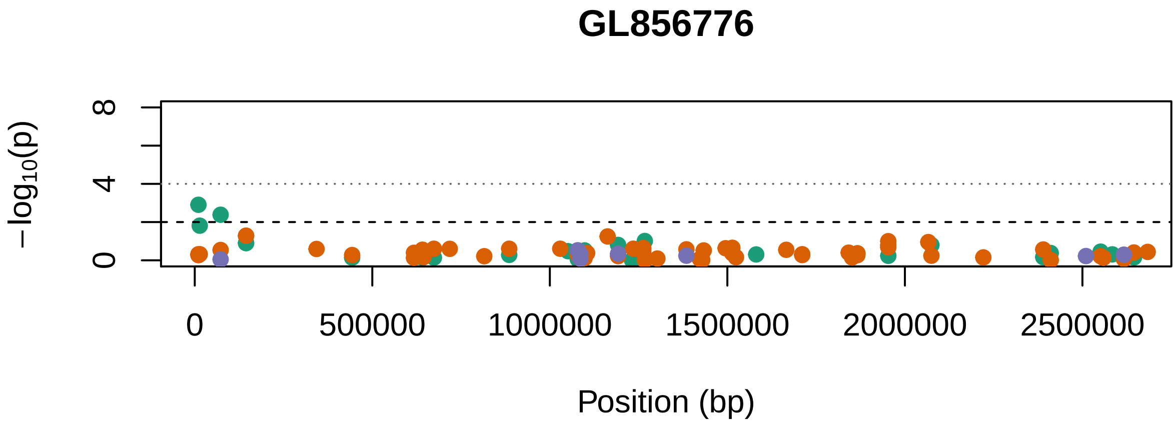
GL856776
−log10(p)
0
4
8
0
500000
1000000
1500000
2000000
2500000
Position (bp)

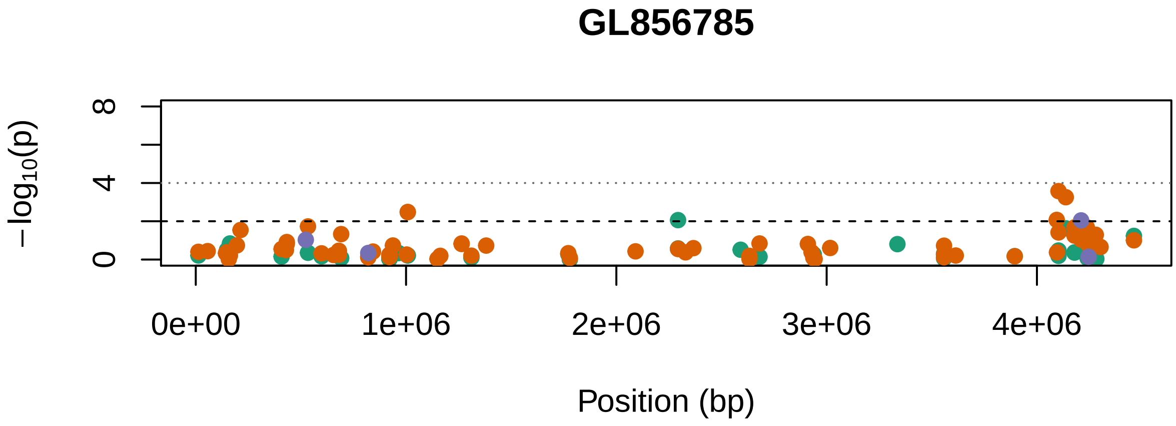
GL856785
−log10(p)
0
4
8
0e+00
1e+06
2e+06
3e+06
4e+06
Position (bp)

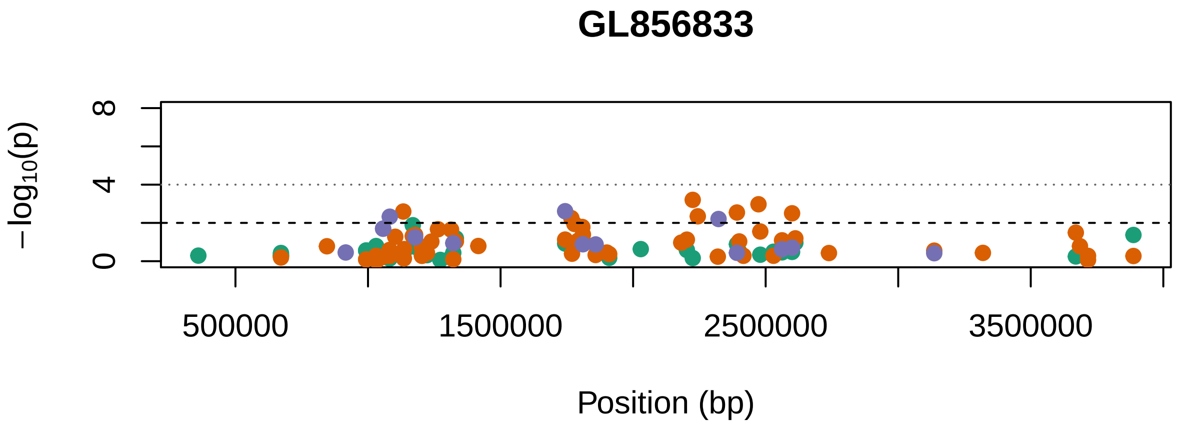
GL856833
−log10(p)
0
4
8
500000
1500000
2500000
3500000
Position (bp)

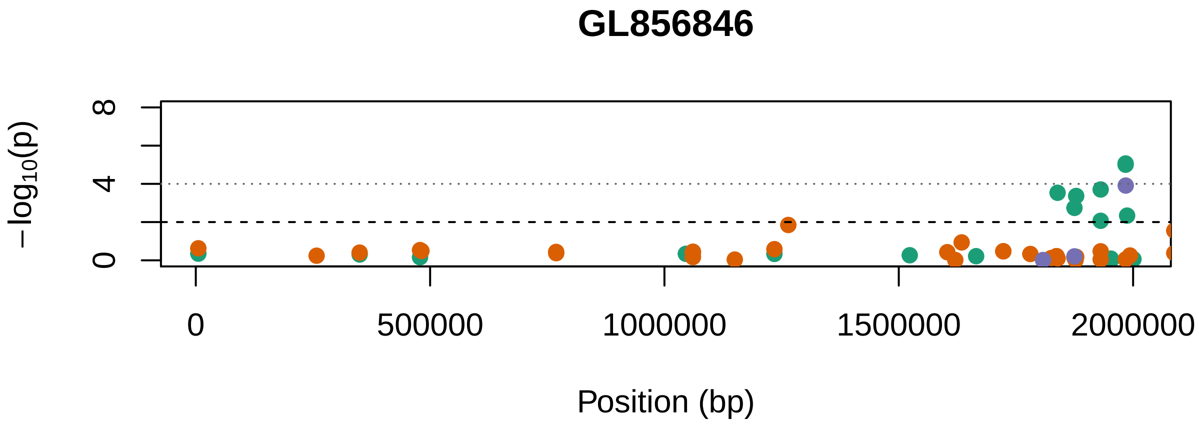
GL856846
−log10(p)
0
4
8
0
500000
1000000
1500000
2000000
Position (bp)

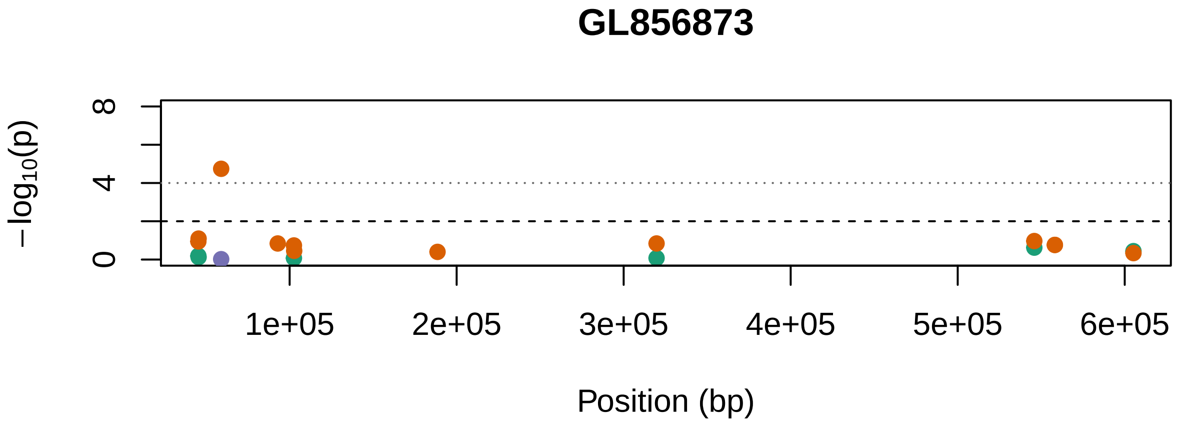
GL856873
−log10(p)
0
4
8
1e+05
2e+05
3e+05
4e+05
5e+05
6e+05
Position (bp)

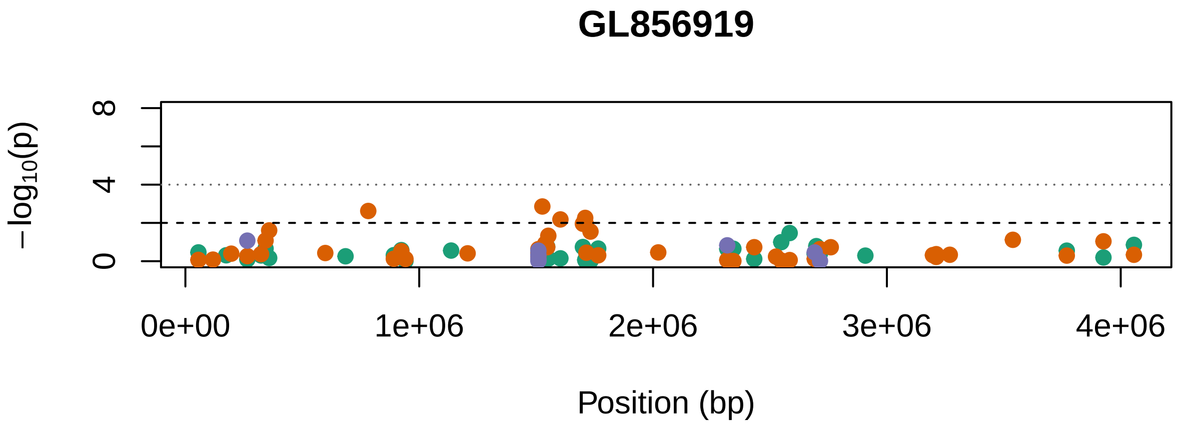
GL856919
−log10(p)
0
4
8
0e+00
1e+06
2e+06
3e+06
4e+06
Position (bp)

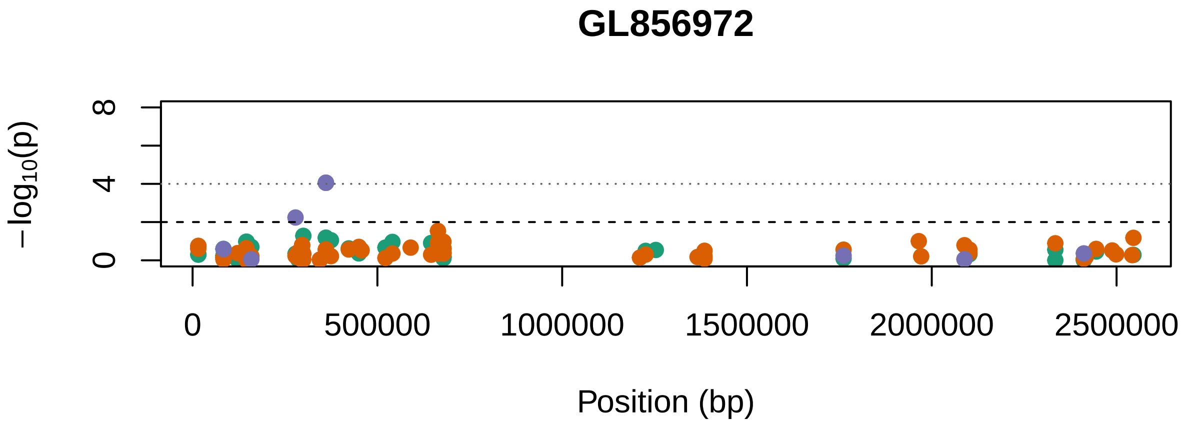
GL856972
−log10(p)
0
4
8
0
500000
1000000
1500000
2000000
2500000
Position (bp)

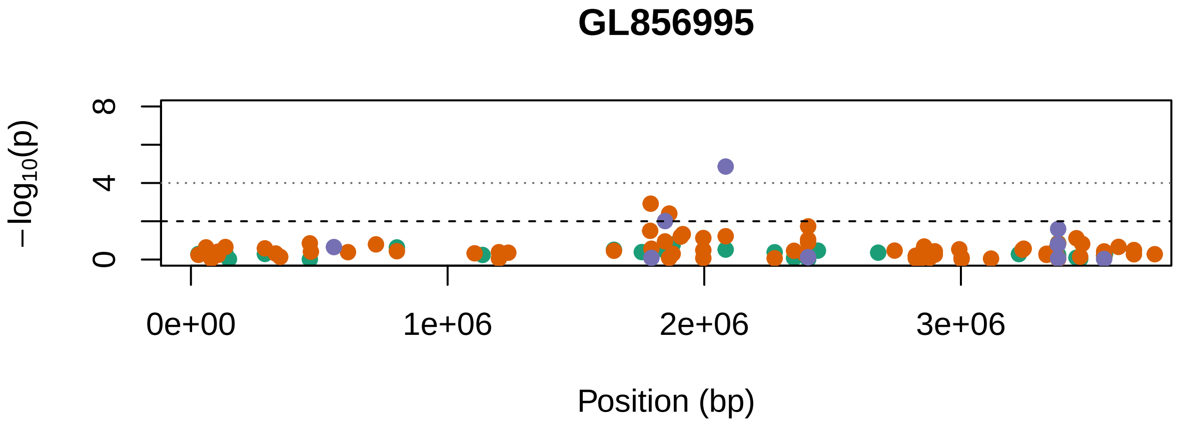
GL856995
−log10(p)
0
4
8
0e+00
1e+06
2e+06
3e+06
Position (bp)

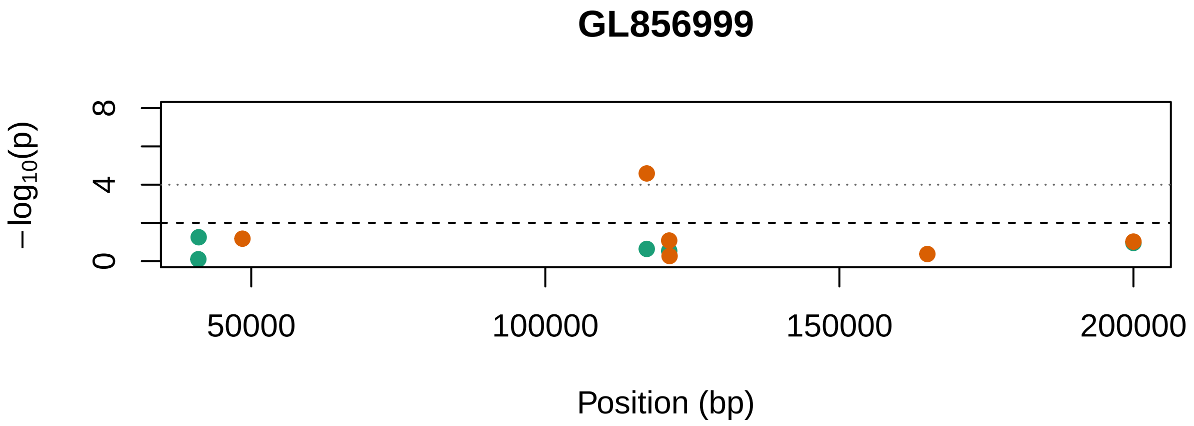
GL856999
−log10(p)
0
4
8
50000
100000
150000
200000
Position (bp)

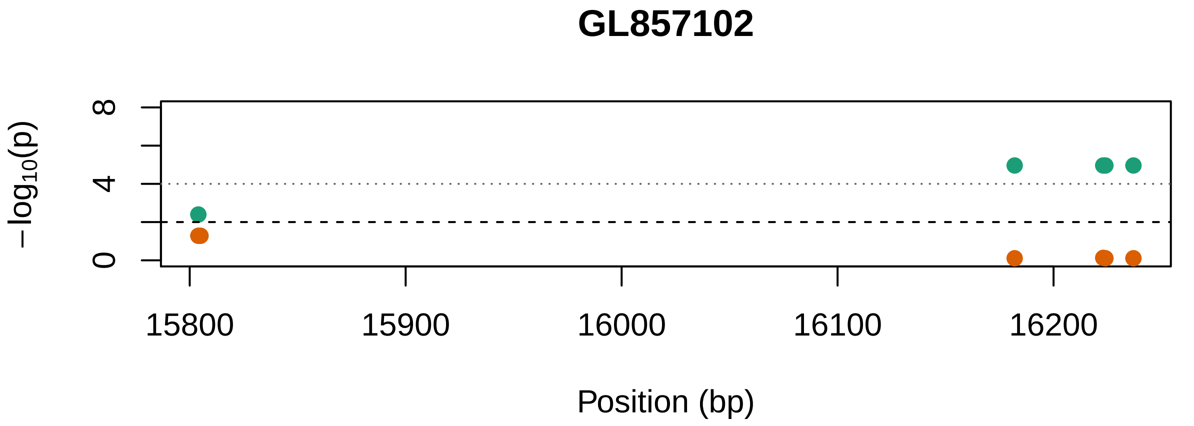
GL857102
−log10(p)
0
4
8
15800
15900
16000
16100
16200
Position (bp)

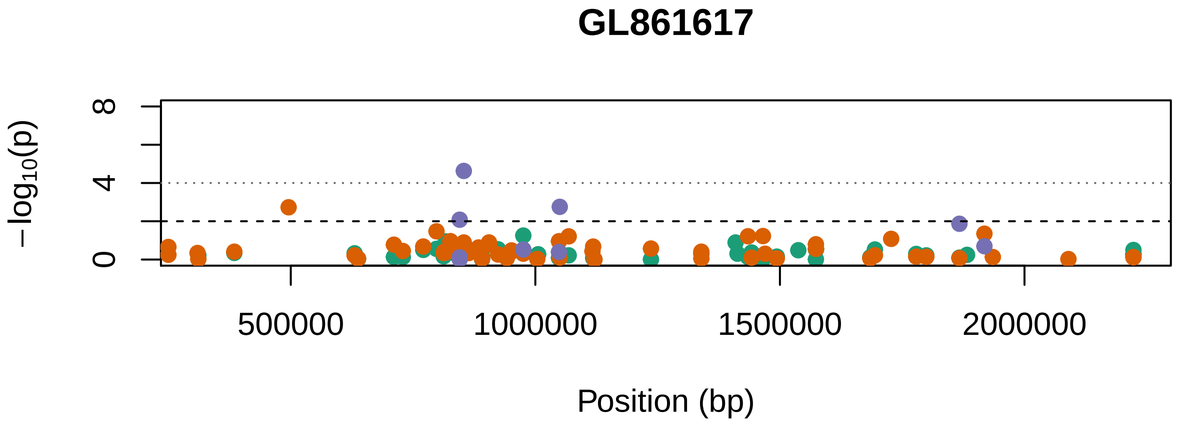
GL861617
−log10(p)
0
4
8
500000
1000000
1500000
2000000
Position (bp)

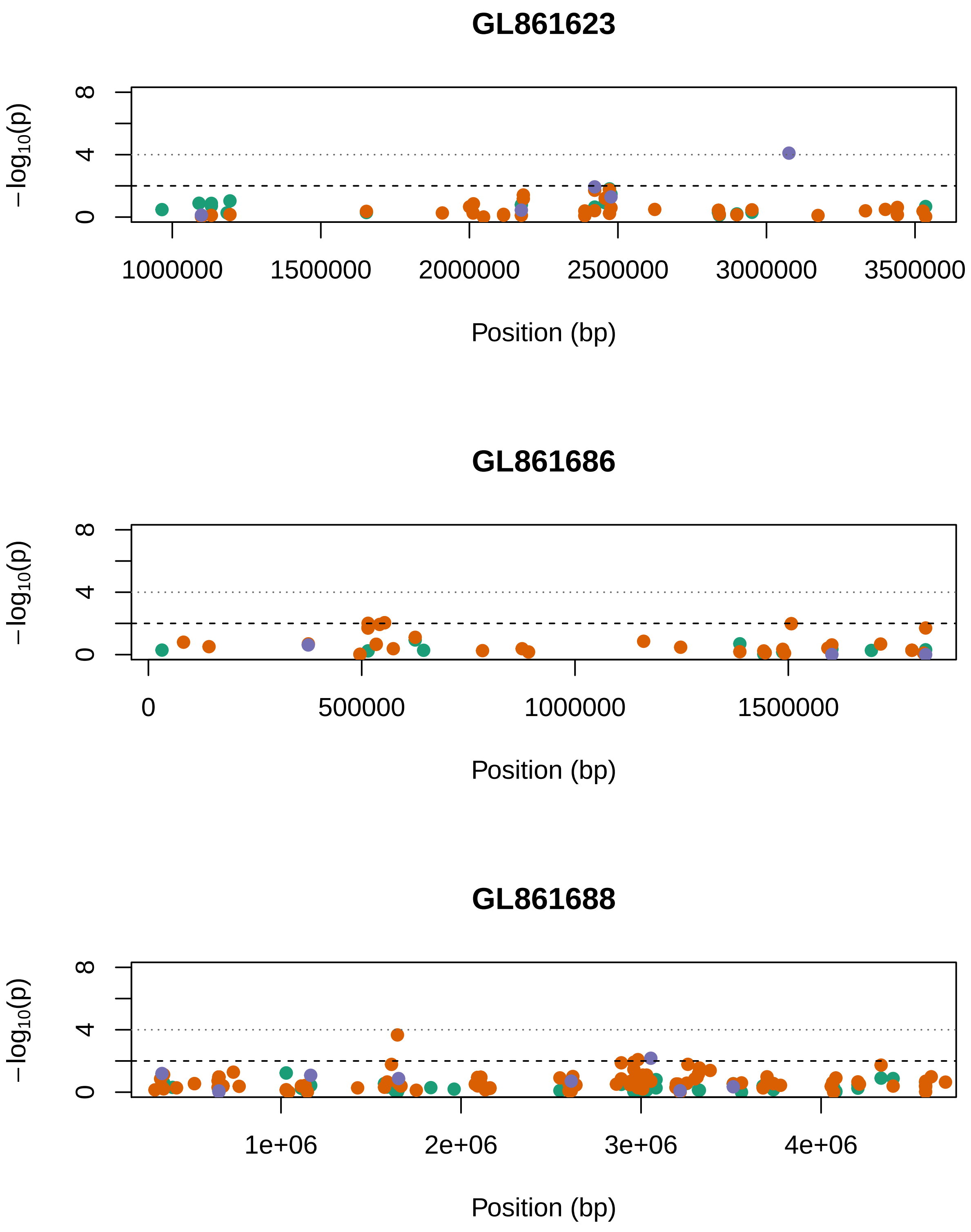
GL861623
8
4
0
−log10(p)
1000000
1500000
2000000
2500000
3000000
3500000
Position (bp)
GL861686
8
4
0
−log10(p)
0
500000
1000000
1500000
Position (bp)
GL861688
8
4
0
−log10(p)
1e+06
2e+06
3e+06
4e+06
Position (bp)

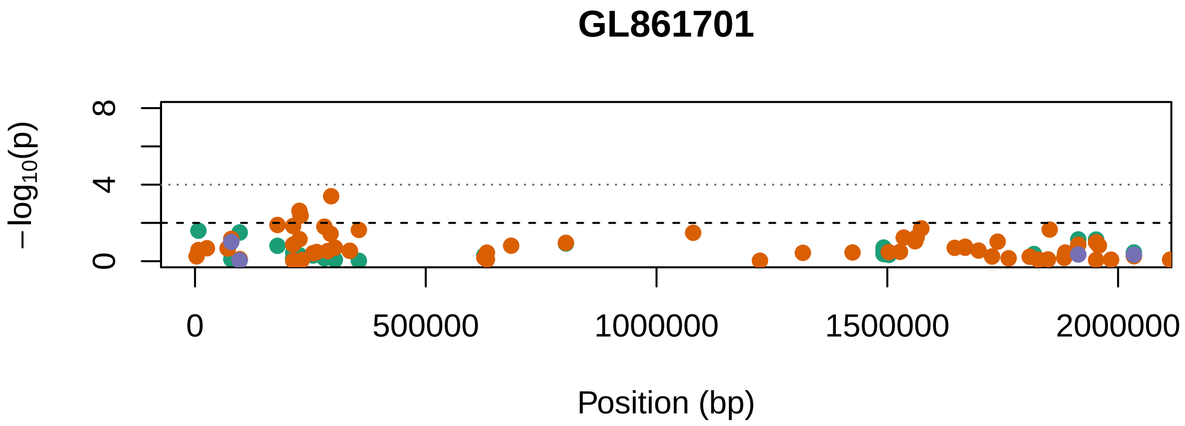
GL861701
−log10(p)
8
4
0
0
500000
1000000
1500000
2000000
Position (bp)

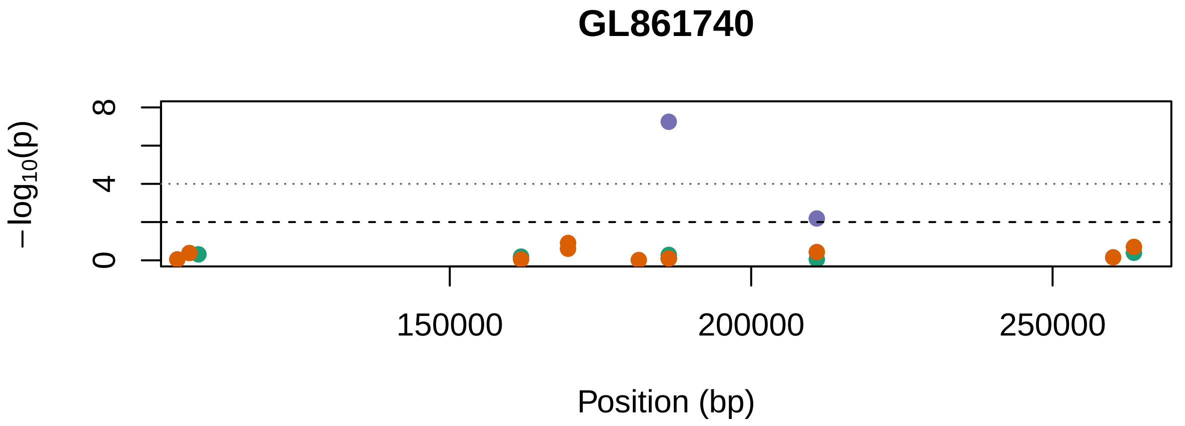
GL861740
−log10(p)
8
4
0
150000
200000
250000
Position (bp)

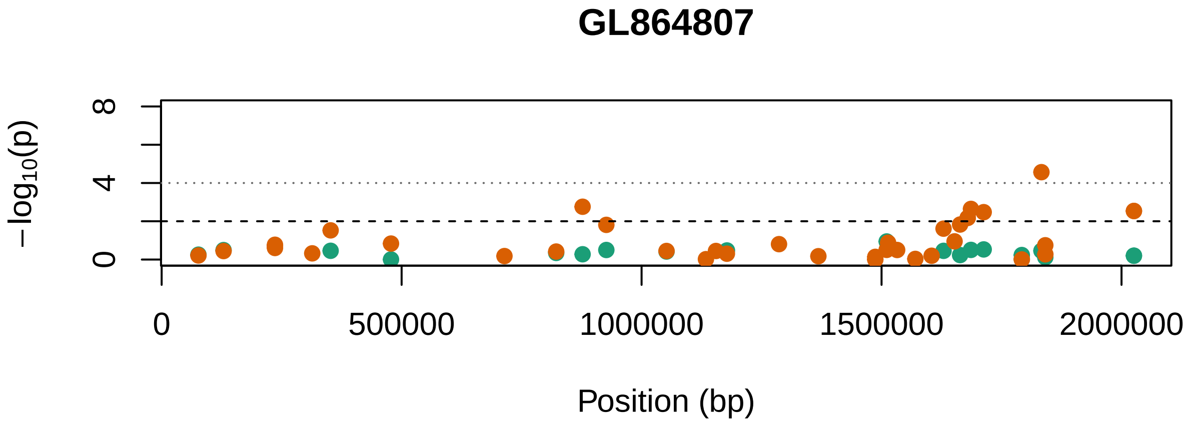
GL864807
−log10(p)
8
4
0
0
500000
1000000
1500000
2000000
Position (bp)

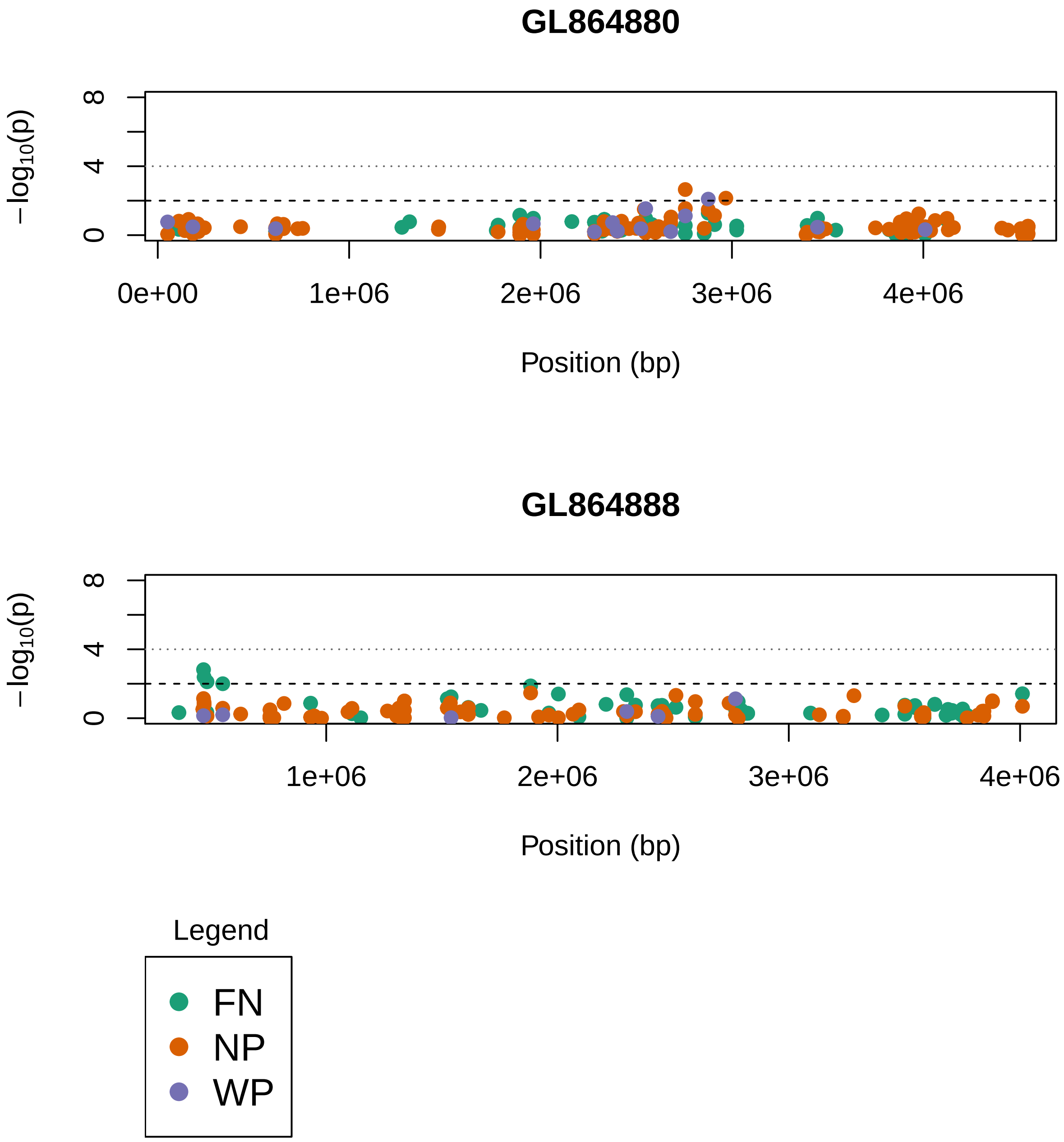

Figure S1. Sixty signatures of selection were identified in 53 scaffolds in the Tasmanian devil genome within 100 kb of a protein coding gene having a human orthologue.

Each SNP was analyzed with *signasel* (https://github.com/hubert-pop/signasel), an R programme that allows detecting selection from genomic time-series (see Methods). To define a signature of selection, we required at least one SNP with a p-value $< 10^{-4}$ (dotted line) or two close SNP with p-values $< 10^{-2}$ (dashed line), corresponding to a FDR of ~ 0.13 [99]. Physical positions (in bp) are relative to the scaffold whose name is reported above the plot. Analyses were performed using the same samples, from three populations (see Table 1), as in [14]. Populations: FN = Freycinet ; NP = Narawntapu ; WP = West Pencil Pine
