# Supplementary figures and images for: Cancer- and behavior-related genes are targeted by selection in the Tasmanian devil (Sarcophilus harrisii)
Source: PLoS One. 2018 Aug 13;13(8):e0201838. doi: 10.1371/journal.pone.0201838 (PMC6089428; doi:10.1371/journal.pone.0201838)

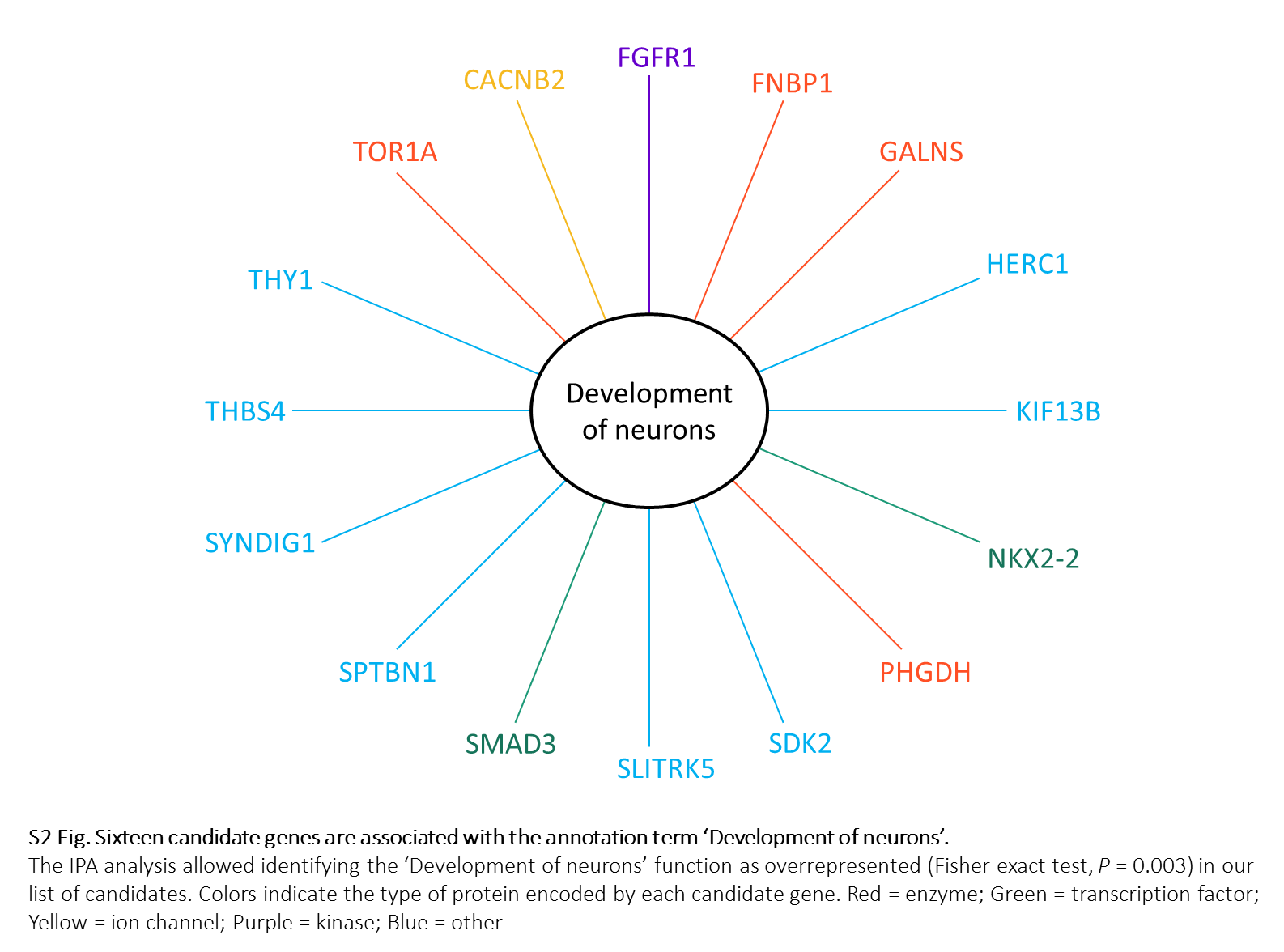

Supplement: S2 Fig — (TIFF) [file pone.0201838.s002.tiff]
